# Swing‐out opening of stromal interaction molecule 1

**DOI:** 10.1002/pro.4571

**Published:** 2023-02-15

**Authors:** Ferdinand Horvath, Sascha Berlansky, Lena Maltan, Herwig Grabmayr, Marc Fahrner, Isabella Derler, Christoph Romanin, Thomas Renger, Heinrich Krobath

**Affiliations:** ^1^ Department for Theoretical Biophysics Johannes Kepler University Linz Linz Austria; ^2^ Institute of Biophysics Johannes Kepler University Linz Linz Austria

**Keywords:** CRAC channels, metadynamics, molecular dynamics, STIM1

## Abstract

Stromal interaction molecule 1 (STIM1) resides in the endoplasmic reticulum (ER) membrane and senses luminal calcium (Ca^2+^) concentration. STIM1 activation involves a large‐scale conformational transition that exposes a STIM1 domain termed “CAD/SOAR”, ‐ which is required for activation of the calcium channel Orai. Under resting cell conditions, STIM1 assumes a quiescent state where CAD/SOAR is suspended in an intramolecular clamp formed by the coiled‐coil 1 domain (CC1) and CAD/SOAR. Here, we present a structural model of the cytosolic part of the STIM1 resting state using molecular docking simulations that take into account previously reported interaction sites between the CC1α1 and CAD/SOAR domains. We corroborate and refine previously reported interdomain coiled‐coil contacts. Based on our model, we provide a detailed analysis of the CC1‐CAD/SOAR binding interface using molecular dynamics simulations. We find a very similar binding interface for a proposed domain‐swapped configuration of STIM1, where the CAD/SOAR domain of one monomer interacts with the CC1α1 domain of another monomer of STIM1. The rich structural and dynamical information obtained from our simulations reveals novel interaction sites such as M244, I409, or E370, which are crucial for STIM1 quiescent state stability. We tested our predictions by electrophysiological and Förster resonance energy transfer experiments on corresponding single‐point mutants. These experiments provide compelling support for the structural model of the STIM1 quiescent state reported here. Based on transitions observed in enhanced‐sampling simulations paired with an analysis of the quiescent STIM1 conformational dynamics, our work offers a first atomistic model for CC1α1‐CAD/SOAR detachment.

## 1 INTRODUCTION

Various cellular processes, such as immune response, gene expression, tumorigenesis, motility, development, astrocyte function, and neuronal signaling, are controlled by elevations in cytosolic Ca^2+^ levels, which in many cases is caused by Store‐Operated Calcium (Ca^2+^) Entry (SOCE; Emrich et al., 2022; Maneshi et al., 2020; Toth et al., 2019; Trebak & Kinet, 2019; Vaeth et al., 2020). A prototypic and well‐studied type of SOCE is regulated by Ca^2+^ release‐activated Ca^2+^ (CRAC) channels, which are activated by the release of Ca^2+^ from the intercellular Ca^2+^ store, the endoplasmic reticulum (ER). CRAC channels are constituted by two proteins: Orai1, a channel protein situated in the plasma membrane (PM), and stromal interaction molecule 1 (STIM1), which has the dual function of sensing ER Ca^2+^ concentration and activating Orai1 channels when ER Ca^2+^ stores are depleted.

In humans, loss‐of‐function mutations of STIM1 lead to severe combined immunodeficiency, autoimmunity, myopathy, and ectodermal dysplasia, whereas gain‐of‐function mutations cause the York and Stormorken syndromes (Online Mendelian Inheritance in Man [OMIM], 2017a, 2017b; OMIM, 2020, 2022; Feske et al., 2006; Lacruz & Feske, 2015). Given this wide‐ranging clinical context, precise understanding of CRAC channel regulation can contribute to the development of immune‐modulating, antiallergic or anticancer drugs (Jairaman & Prakriya, 2013; Malli & Graier, 2017; Vashisht et al., 2015).

CRAC channels are regulated by a subtle balance between the STIM1 active and quiescent states. STIM1 is a single‐pass *trans*‐ER‐membrane protein with a luminal and a cytosolic domain. When ER Ca^2+^ stores are full, Ca^2+^ ions bind to the STIM1 EF‐hand domain in the ER lumen, stabilizing the STIM1 quiescent state (Zheng et al., 2008). Upon store depletion, STIM1‐bound Ca^2+^ ions dissociate and trigger a conformational change that is conveyed across the ER membrane towards the cytoplasmic domain of STIM1 (Fahrner et al., 2018; Jennette et al., 2022; Liou et al., 2005; Muik et al., 2011; Shim et al., 2015). This cytosolic portion is composed of three coiled‐coil domains, CC1, CC2, and CC3, and a polybasic domain at the C‐terminus. CC1 consists of three alpha helices named CC1α1–3. CC2 and CC3 are jointly called “CRAC activation domain” (CAD) or “STIM‐Orai‐Activating Region” (SOAR). CC1α1 is critical in maintaining the STIM1 quiescent state since it binds the CAD/SOAR domain, thus keeping it sequestered and preventing it from binding to and opening the Orai1 channel under resting conditions. Upon ER Ca^2+^ store depletion, this “autoinhibitory clamp” is released, which leads to homomerization and elongation of STIM1 CC1. Furthermore, in a large‐scale reorientation, CAD/SOAR is rotated away from the ER membrane and extended towards the PM, allowing it to bind to Orai1, opening the CRAC channel, and triggering Ca^2+^ entry into the cell (Derler et al., 2016; Lewis, 2019).

Given the crucial role of the CC1‐CAD/SOAR clamp within the larger context of CRAC channel regulation, detailed understanding of CC1α1‐CAD/SOAR binding is of critical importance. Previous studies could identify several sites in CC1α1 as key components of the CC1α1‐CAD/SOAR clamp (Ma et al., 2015; Muik et al., 2011; van Dorp et al., 2021; Zhou et al., 2013). So far, these studies have been impeded by the lack of detailed structural information about the STIM1 active or quiescent states. While several fragments of the STIM1 cytosolic domain could be resolved in nuclear magnetic resonance (NMR) and x‐ray crystallography experiments (Cui et al., 2013; Rathner et al., 2021; Stathopulos et al., 2013; Yang et al., 2012), it is still unclear how they relate to the full‐length protein under physiological conditions. The first detailed description of STIM1 dimeric conformation under near‐physiological conditions was reported in a seminal publication by van Dorp et al. (2021) There, the authors used single‐molecule Förster resonance energy transfer (smFRET) measurements on the cytosolic domain of STIM1 to infer that CC1α1‐CAD/SOAR binding competes with CC1α1‐CAD/SOAR' binding. That is, in their experiments CAD/SOAR primarily bound to the CC1α1 helix of the opposite monomer (denoted by a prime), resulting in a domain‐swapped configuration (Bennett et al., 1994; Rousseau et al., 2003).

The present study underpins and expands upon previous attempts at determining the STIM1 structure with a detailed molecular model of the cytosolic STIM1 quiescent state that encompasses all previously known CC1α1‐CAD/SOAR interaction sites. Based on our model, we carried out conventional and enhanced‐sampling molecular dynamics (MD) simulations to study the interactions underlying the CC1α1‐CAD/SOAR clamp with atomistic resolution. Our model was tested by inferring key mutation sites from simulations deliberately targeted at disrupting CC1α1‐CAD/SOAR binding and constitutively activating STIM1. Based on these model predictions, we performed whole‐cell electrophysiology and FRET experiments. Finally, our model is extended to a STIM1 dimer embedded in a model ER membrane, which corroborates results obtained from our monomer and suggests a novel mechanistic model for how STIM1 switches from its quiescent to its active, elongated state.

## 2 RESULTS

### 2.1 Structural model of the STIM1 quiescent state

**FIGURE 1:**
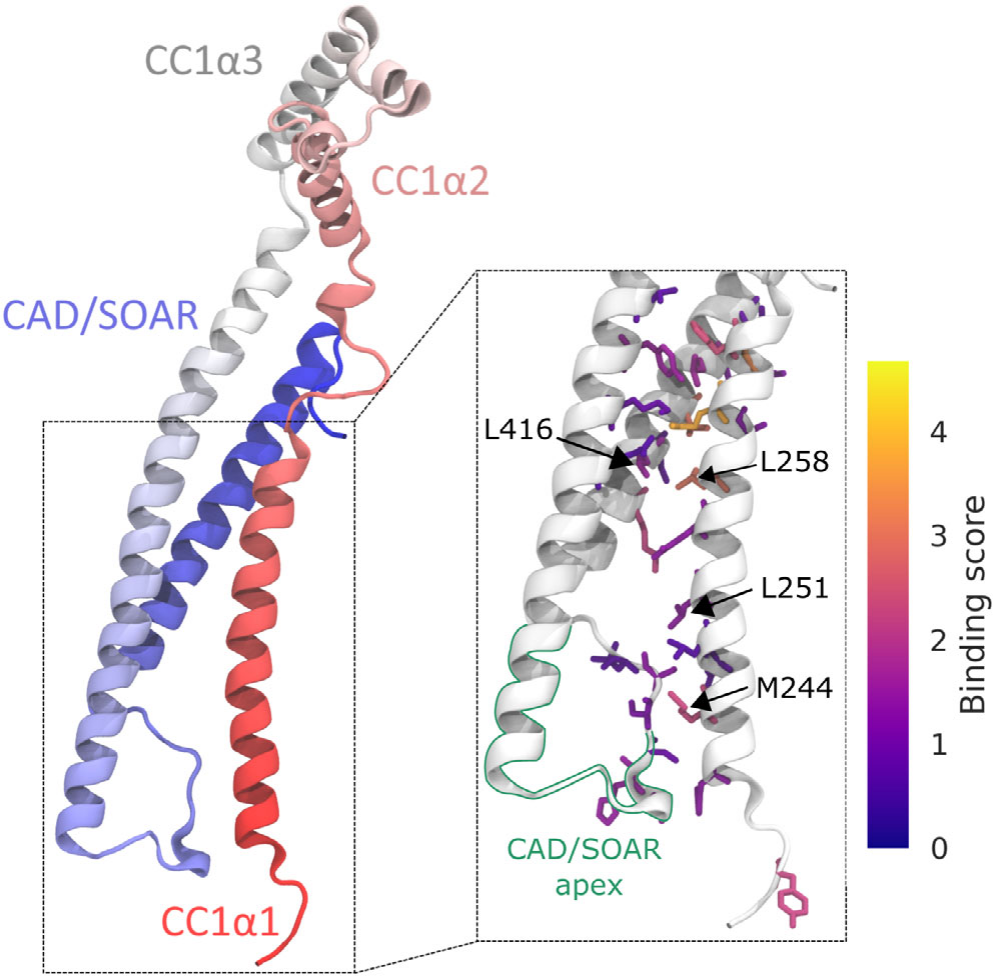
Model structure of the stromal interaction molecule (STIM) 1 quiescent state (residues 234–443). The N‐terminus is shown in red, the C‐terminus in blue. The zoom‐in in the right panel highlights the 30 most important CC1α1‐CAD/SOAR interface residues, color‐coded with their respective binding scores *S*_*i*_. The CAD/SOAR apex is outlined in green.

To obtain an initial conformation for the CC1–CC3 clamp, we performed docking simulations of CC1α1 (PDB id 6YEL; Rathner et al., 2021) and CAD/SOAR (PDB id 3TEQ; Yang et al., 2012). CC1α1‐CAD/SOAR binding sites identified in references (Ma et al., 2015; van Dorp et al., 2021) were used to define restraints for the docking simulations (see Section 4.1). We discarded output clusters that entailed severe clashes between CAD/SOAR and the ER membrane, which was not explicitly accounted for in the docking. Of the remaining candidates, the structure with the best docking score was selected. This docked CC1α1‐CAD/SOAR model still lacked helices CC1α2 and CC1α3. These elements were modeled based on the compactly packed dimer in the CC1 NMR model (PDB id 6YEL) and manually joined to the docked CC1‐CAD/SOAR fragments. After equilibration, we obtained a model of STIM1 in its quiescent state comprising residues 234–443 (Figure 1 and File S1).

### 2.2 Identification of key residues constituting the CC1α1‐CAD/SOAR clamp

While the construction of our model structure required only a small number of known CC1α1‐CAD/SOAR interaction sites, our model allowed us to study the full CC1α1‐CAD/SOAR binding interface. We find that the CC1α1‐CAD/SOAR clamp is primarily facilitated by the CAD/SOAR apex (residues 392–405) binding to the N‐terminal part of CC1α1, as well as residues 255–268 in CC1α1 binding to residues 355–378 and 413–427 in CAD/SOAR (see Figure S1). We designed a binding score *S*_*i*_ that reflects how many contacts an interface residue *i* forms with the opposing domain and how stable these contacts are. Using this score, we were able to discern which amino acids are of key importance for CC1α1‐CAD/SOAR binding and, thereby, for balancing the equilibrium of STIM1 activation/inactivation (see Figures 1 [zoom‐in], 2, and Movie S1). The score *S*_*i*_ is defined as $S_{i}=\sum_{j}\omega_{\mathrm{ij}}$, where *ω*_*ij*_ denotes the contact frequency for contacts between two binding residues *i*, *j* (see Section 4.3). In a similar manner, we identified the most important CC1α1‐CAD/SOAR binding partners by sorting pairs of residues by their contact frequency (see Table S1).

**FIGURE 2:**
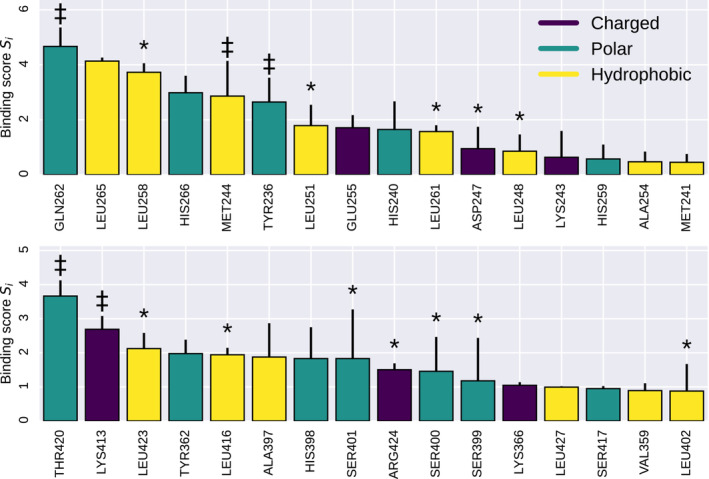
Binding scores *S*_*i*_ calculated for the wild type. Error bars denote the standard deviation taken over 12 independent metadynamics runs. The top and bottom panels show residues in CC1α1 and CAD/SOAR, respectively. Positions marked with an asterisk (*) refer to known binding residues (Butorac et al., 2019; Hirve et al., 2018; Ma et al., 2015; Muik et al., 2011). Positions marked with a dagger ‡ are novel binding residues that are here also investigated experimentally.

Several known interface residues, such as D247, L248, L251, L258, L261, L416, or L423 (Hirve et al., 2018; Ma et al., 2015; Muik et al., 2011), feature among our top‐scoring binding residues. In addition, our score *S*_*i*_ predicts a set of novel binding residues such as Y236, M244, Q262, E370, A397, I409, or T420 (Figure 2).

### 2.3 Free energy of CC1α1‐CAD/SOAR unbinding reflects CRAC channel currents in resting cells

To scrutinize the predictive power of our structural model, we carried out well‐tempered metadynamics simulations (Barducci et al., 2008). These simulations allowed us to both enhance the sampling of possible CC1α1‐CAD/SOAR binding configurations and to quantify the strength of CC1α1‐CAD/SOAR binding. The metadynamics bias was applied to two collective variables: the CC1α1‐CAD/SOAR center of mass distance and number of contacts, respectively (see Section 4.3). Metadynamics facilitates the crossing of free energy barriers by introducing a time‐dependent bias potential that successively counter‐balances free energy minima. By completely balancing out the free energy minimum corresponding to the CC1α1‐CAD/SOAR bound state and detaching CAD/SOAR from CC1α1, we calculate the free energy of CC1α1‐CAD/SOAR unbinding, Δ*G*_unb_.

We performed simulations on mutants of 12 different sites to investigate how they affect CC1α1‐CAD/SOAR binding (Figure S2). Of the mutated positions, eight were picked from our list of top‐scoring CC1α1‐CAD/SOAR binding residues (Y236, M244, L251, Q262, A397, K413, L416, and T420). Among those, L251 and L416 are known binding residues (Muik et al., 2011), the others were newly identified in this study. In addition, we selected position I409 for its deep embedding in the CAD/SOAR hydrophobic core, allowing for effective disruption of the CC1α1‐CAD/SOAR clamp. Since I409 primarily contributes to hydrophobic binding of CC2 and CC3, it has a low CC1α1‐CAD/SOAR binding score and therefore does not appear in Figure 2. To complement our set of charged mutations, we selected position E370, which forms a CC1α1‐CAD/SOAR salt bridge (Figure S3a,b). At these positions, we introduced point mutations tailored towards disrupting CC1α1‐CAD/SOAR binding (see Section 4.5 for details). To check the case of putative enhanced CC1α1‐CAD/SOAR binding, we also performed simulations of the R426L mutant, which is known to stabilize the STIM1 quiescent state (Fahrner et al., 2014; Ma et al., 2020; Muik et al., 2011). The neutral mutation K285A was selected as a control. Since K285 is not involved in CC1α1‐CAD/SOAR binding, its binding score is zero.

For each of these mutants, we performed extensive metadynamics simulations to calculate Δ*G*_unb_ as well as binding scores *S*_*i*_. Complementing our simulated results, we carried out whole‐cell patch clamp electrophysiology experiments in HEK293 cells to experimentally characterize the impact of mutations in STIM1 intramolecular interaction sites on CRAC channel function. These experiments track the current density of Orai1 channels over time. In the case of STIM1 wildtype (WT), the current density is ~0 at the start of the recording, *I*(*t* = 0) = 0 pA/pF, and gradually increases upon passive store‐depletion as STIM1 proteins switch from their quiescent to their extended states, cumulatively activating Orai1 channels.

**FIGURE 3:**
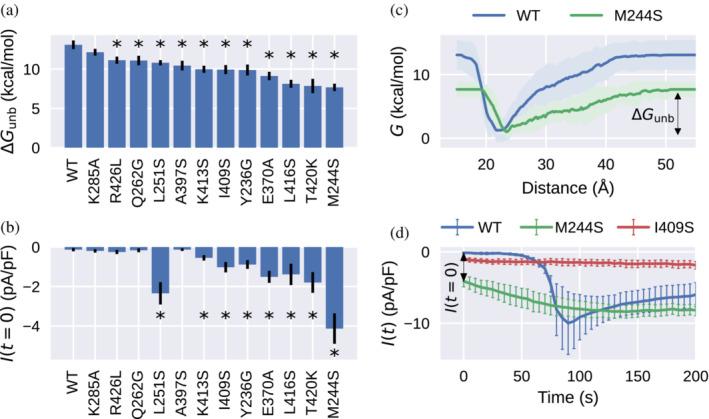
(a) Free energies of CC1α1‐CAD/SOAR unbinding, Δ*G*_unb_, for the wild type (WT) and different STIM1 mutants. (b) Current density before store depletion, *I* (*t* = 0), measured in the patch clamp experiment. Error bars in (a) and (b) indicate the standard error of the mean over several independent metadynamics runs or patch clamp experiments, respectively. (c) Free energy profiles for the WT and an exemplary constitutively active mutant, M244S. (d) Time course of whole‐cell inward currents activated by passive endoplasmic reticulum Ca^2+^ store depletion of HEK293 cells co‐expressing Orai1 WT together with the STIM1 WT or M244S or I409S mutants, respectively. Asterisks (*) indicate statistical significance with *p*‐value < 0.05 with respect to the WT.

Figure 3 condenses the central results from our metadynamics simulations and patch clamp experiments on the STIM1 WT and 12 different STIM1 mutants. For all tested mutants, we calculated Δ*G*_unb_. For almost all tested mutants, current densities measured before ER Ca^2+^ store depletion, *I* (*t* = 0), reflect the reduced Δ*G*_unb_, indicating a destabilization of the STIM1 quiescent state prior to ER Ca^2+^ store depletion (Figure 3a,b). Thus, STIM1 binds to Orai1 already under resting conditions, which facilitates Ca^2+^ entry. Figure 3c shows the free energy *G* as a function of CC1α1‐CAD/SOAR distance for the STIM1 WT and the exemplary M244S mutant, respectively. Figure 3d illustrates time series of Orai1 current densities in whole‐cell patch clamp experiments for the STIM1 WT and two exemplary mutants, M244S and I409S (for data on further mutants, see Figures S4–S6). Each of the 12 simulated and measured mutants is discussed below in detail.

### 2.4 Experimental and simulated characterization of key CC1α1‐CAD/SOAR interaction residues

Mutants such as L251S or L416S have been shown to constitutively activate STIM1 by disrupting CC1α1‐CAD/SOAR binding, destabilizing the STIM1 quiescent state, and activating Orai1 (Muik et al., 2011). This mechanistic interpretation is corroborated by our simulations, which show that the strength of CC1α1‐CAD/SOAR binding is lowered by about 2.3 kcal/mol (13%) or 5 kcal/mol (38%) in the L251S and L416S mutants, respectively, compared with the WT (Figure 3a,C). Notably, our simulations show that L251S disrupts binding between CC1α1 and the CAD/SOAR apex, which is an immediate neighbor of position 251 (Figure 1).

A number of key hydrophobic CC1‐CAD/SOAR interaction sites have been identified in previous studies (Butorac et al., 2019; Hirve et al., 2018; Ma et al., 2015; Muik et al., 2011; van Dorp et al., 2021). Our simulations complement this list with two additional hydrophobic sites, M244 and I409. M244S had an especially drastic effect on simulated CC1α1‐CAD/SOAR binding, yielding the lowest Δ*G*_unb_ of all mutants and strongly broadening and flattening the free energy profile (Figures S4 and S7). Looking at mutation‐induced changes in the binding score *S*_*i*_, this can clearly be attributed to disrupted binding of CC1α1 and the CAD/SOAR apex (Figure S8). I409S similarly reduced Δ*G*_unb_. In line with reduced Δ*G*_unb_, we found that the mutations M244S and I409S resulted in constitutive Orai1 currents (Figure 3D). When compared with other constitutively active mutants, STIM1 I409S elicited only small current densities. This behavior, together with diminished STIM1–Orai1 colocalization (Figure S9), indicates that I409 is involved in the binding to and activation of Orai1 channels. Summarizing, we find that hydrophobic‐to‐polar substitutions L251S, L416S, and M244S primarily affect the binding of the CAD/SOAR apex to CC1α1, resulting in constitutive Orai1 currents; I409S similarly elicited constitutive Orai1 activation but additionally interfered with STIM1–Orai1 interaction.

Another hydrophobic residue among our top‐scoring binding residues in CAD/SOAR is A397 (Figure 2). Surprisingly, although A397S resulted in clear lowering of CC1α1‐CAD/SOAR binding strength Δ*G*_unb_, A397S did not affect store‐operated STIM1 function in the patch clamp experiment (Figures 3a and S5). We assume that this discrepancy points to a limitation of our water‐solvated STIM1 model. We will return to the case of A397S in the context of a more comprehensive dimeric STIM1 model in Section 2.6.

Besides hydrophobic interactions, our simulations indicate that CC1α1‐CAD/SOAR binding is stabilized to a considerable degree by electrostatic attraction between oppositely charged residues (salt bridges). Specifically, we found that Δ*G*_unb_ was significantly reduced by the mutations E370A (in CC2) and K413S (in CC3). Our model indicates that these residues form salt bridges with protonated H259 and E255 in CC1α1, respectively (Figure S3), both of which feature among our top‐scoring binding residues (Figure 2). Note that E370 is assigned a rather low binding score of *S*_*i*_ = 0.7 which suggests that our score underestimates electrostatic binding contributions in this case. In line with the impact on the CC1α1‐CAD/SOAR binding interface, STIM1 E370A and STIM1 K413S led to constitutive activation of Orai1 currents which further increased after passive store‐depletion to WT‐like levels. In addition, STIM1 E370K enhanced constitutive and maximal current levels even further. These results indicate, for the first time, that electrostatic CC1α1‐CAD/SOAR interactions are a necessary prerequisite for maintaining the STIM1 quiescent state.

Our binding score shows that several hydrophilic residues contribute to CC1α1‐CAD/SOAR binding (Figure 2). We simulated Q262G, Y236G, and T420K, all of which led to a reduction of Δ*G*_unb_ (Figure 3a). Whereas Q262G retained store‐operated activation of Orai1 in the patch clamp experiment similar to the WT, Q262K was constitutively active (Figure S5). Y236G accelerated the store‐operated activation of Orai1 compared with the WT, indicating a lowered kinetic barrier. Consistent with our model predictions, T420K was constitutively active (Figure S5). Thus, we find that CC1α1‐CAD/SOAR binding is facilitated not only by the hydrophobic effect and electrostatic interactions between charged residues, but also by electrostatic interactions between polar groups, the disruption of which constitutively activates STIM1.

The R426L mutation was previously found to significantly reduce store‐operated STIM1 activation (Fahrner et al., 2014; Ma et al., 2020; Muik et al., 2011). Surprisingly, we found that this mutation also leads to slightly reduced CC1α1‐CAD/SOAR binding strength Δ*G*_unb_ as compared with the WT. We are therefore led to assume that the observed reduction in Orai1 currents is not due to a change in CC1α1‐CAD/SOAR interaction but to some other effect that destabilizes the STIM1 active state. We will return to the case of R426L below in Section 2.6.

As a control, we simulated the K285A mutant, which behaves similar to the WT in the patch clamp experiment (Figure S5). Indeed, out of all mutants simulated, K285A is the only one where Δ*G*_unb_ is statistically indistinguishable from the WT (Figure 3a).

In line with their weakened CC1α1‐CAD/SOAR binding, we found that in all constitutively active STIM1 mutants the free energy minimum corresponding to the bound state was broadened, resulting in a more structurally diverse ensemble of closed STIM1 conformations (Figure S7). In addition to the mutations discussed here, we tested point‐substitutions at several positions with low binding scores *S*_*i*_ (ranging from 0.01 to 0.62). As expected, these mutations preserved STIM1 store‐operated function in the patch clamp experiment, which indicates that low binding scores faithfully reflect reduced importance for the STIM1 autoinhibitory clamp (Figure S6).

Summarizing, our analysis of mutations introduced at key binding sites reveals that weakened CC1α1‐CAD/SOAR binding particularly involves a disruption of binding between the CAD/SOAR apex and the CC1α1 N‐terminus (M244S, L251S, and L416S). Besides the CAD/SOAR apex, a “hinge region” near the CC1α1 C‐terminus (formed, e.g., by hydrophilic Q262 and T420) proves crucial for anchoring CAD/SOAR and CC1α1. Overall, the CC1α1‐CAD/SOAR inhibitory clamp is stabilized by various interactions, including hydrophobic and polar ones as well as salt bridges.

### 2.5 MD‐derived mutations trigger STIM1 homomerization and C‐terminal extension in live‐cell FRET experiments

**FIGURE 4:**
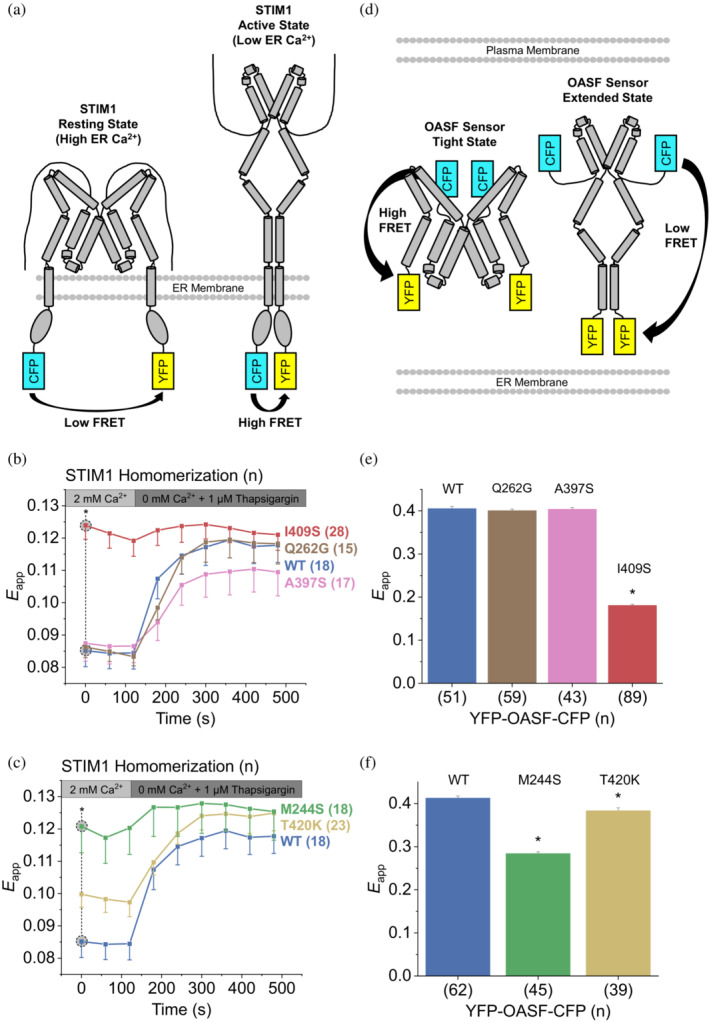
(a) Schematic representation of STIM1 homomerization. Upon STIM1 activation elicited by Ca^2+^ store depletion, the N‐termini in the endoplasmic reticulum (ER) lumen and the attached fluorophores (CFP and YFP; cyan and yellow rectangles) come into closer proximity, leading to increased FRET transfer efficiency. (b,c) Homomerization experiments of N‐terminally tagged CFP‐STIM1/YFP‐STIM1 mutants showing the change in intermolecular Förster resonance energy transfer (FRET) efficiency (*E*_app_) elicited by ER Ca^2+^ store depletion. (D) YFP‐OASF‐CFP conformational sensor. In the tight state, the two fluorophores CFP and YFP (cyan and yellow rectangles) are in close proximity, which translates to high FRET efficiency and vice versa for the extended state. (e,f) Intramolecular FRET efficiency (*E*_app_) showing conformational changes of YFP‐OASF‐CFP mutants. Asterisks (*) indicate statistical significance with *p*‐value < 0.05. Experiments were replicated on at least two different days using independent transfections with the indicated number of cells (*n*). Data represent mean values ± SEM. Panels (b,c) as well as (e,f) are equivalent but split for ease of legibility.

To further corroborate our metadynamics simulations and patch clamp experiments on the STIM1 protein level, we performed STIM1 homomerization experiments for selected mutations in HEK293 cells co‐expressing STIM1 constructs N‐terminally tagged with CFP or YFP (cyan/yellow fluorescent protein, Figure 4a). For this, the change in intermolecular FRET efficiency (*E*_app_) upon Ca^2+^ store depletion elicited by 1 μM thapsigargin was measured over several minutes. Our data reveal a characteristic rise of the intermolecular *E*_app_ for STIM1 WT, which is elicited by the homomerization of STIM1 activated by empty Ca^2+^ stores (Figure 4b,c). In line with lowered Δ*G*_unb_ as well as constitutive Orai1 activity observed in our patch clamp experiments, STIM1 M244S as well as I409S were already maximally activated under resting conditions and did not show a significant increase in FRET efficiency upon Ca^2+^ store depletion (Figure 4b,c). The baseline FRET efficiency of STIM1 T420K was higher than that of the WT, but it still showed a distinct FRET increase indicative of Ca^2+^ store dependence (Figure 4c). The behavior of STIM1 Q262G and A397S did not significantly differ from the WT (Figure 4b), which is in agreement with our patch clamp results.

As a further means of comparison between simulation and experiment, we performed intramolecular FRET experiments using the C‐terminal orai‐activating small fragment (“OASF”, aa 233–474) sensor construct. This double‐fluorescently labeled, soluble fragment of STIM1 acts as a potent conformational sensor (denoted as YFP‐OASF‐CFP, Figure 4d) and provides a direct counterpart to the simulated model structure of the STIM1 quiescent state (aa 234–443, Figure 1). YFP‐OASF‐CFP WT exhibited high intramolecular *E*_app_ consistent with the quiescent, tight state of STIM1 (Figure 4e,f; Fahrner et al., 2014; Muik et al., 2011). In line with our data presented above, YFP‐OASF‐CFP M244S and I409S both showed a significant decrease of intramolecular *E*_app_ that is characteristic for a switch of the conformational sensor into an extended, activated state (Figure 4e,f). The FRET efficiency of YFP‐OASF‐CFP T420K was higher than that of M244S and I409S, but still significantly reduced with respect to the WT (Figure 4f). YFP‐OASF‐CFP Q262G and A397S did not significantly alter the intramolecular FRET efficiency of the sensor construct, which fits our homomerization and patch‐clamp results. To summarize, FRET experiments monitoring the homomerization or conformational switch of STIM1 corroborate the predicted activating effect of mutations M244S, I409S, whereas T420K had a reduced but significant activating effect.

Comparing the results obtained from our MD simulations and FRET measurements, we note several points of divergence, such as the limited effect of T420K on YFP‐OASF‐CFP *E*_app_ (Figure 4f) contrasted with the very large T420 binding score (Figure 2), or the A397S mutant, which reduces Δ*G*_unb_ in the simulation (Figure 3a) but shows no impact on STIM1 homomerization or the OASF conformational sensor (Figure 4b,e). These points highlight that our STIM1 model provides insights into only one facet of the STIM1 activation cascade, but does not capture aspects such as STIM1 dimerization or higher‐order oligomerization, STIM1/Orai1 interaction with auxiliary proteins, STIM1 C‐terminal extension or STIM1 binding to or gating of Orai1 channels (Berlansky et al., 2021; Hogan et al., 2010; Muik et al., 2011). To alleviate some of these limitations, we constructed a more comprehensive STIM1 model consisting of a STIM1 dimer embedded in a model ER membrane.

### 2.6 STIM1 dynamics in a dimeric model

**FIGURE 5:**
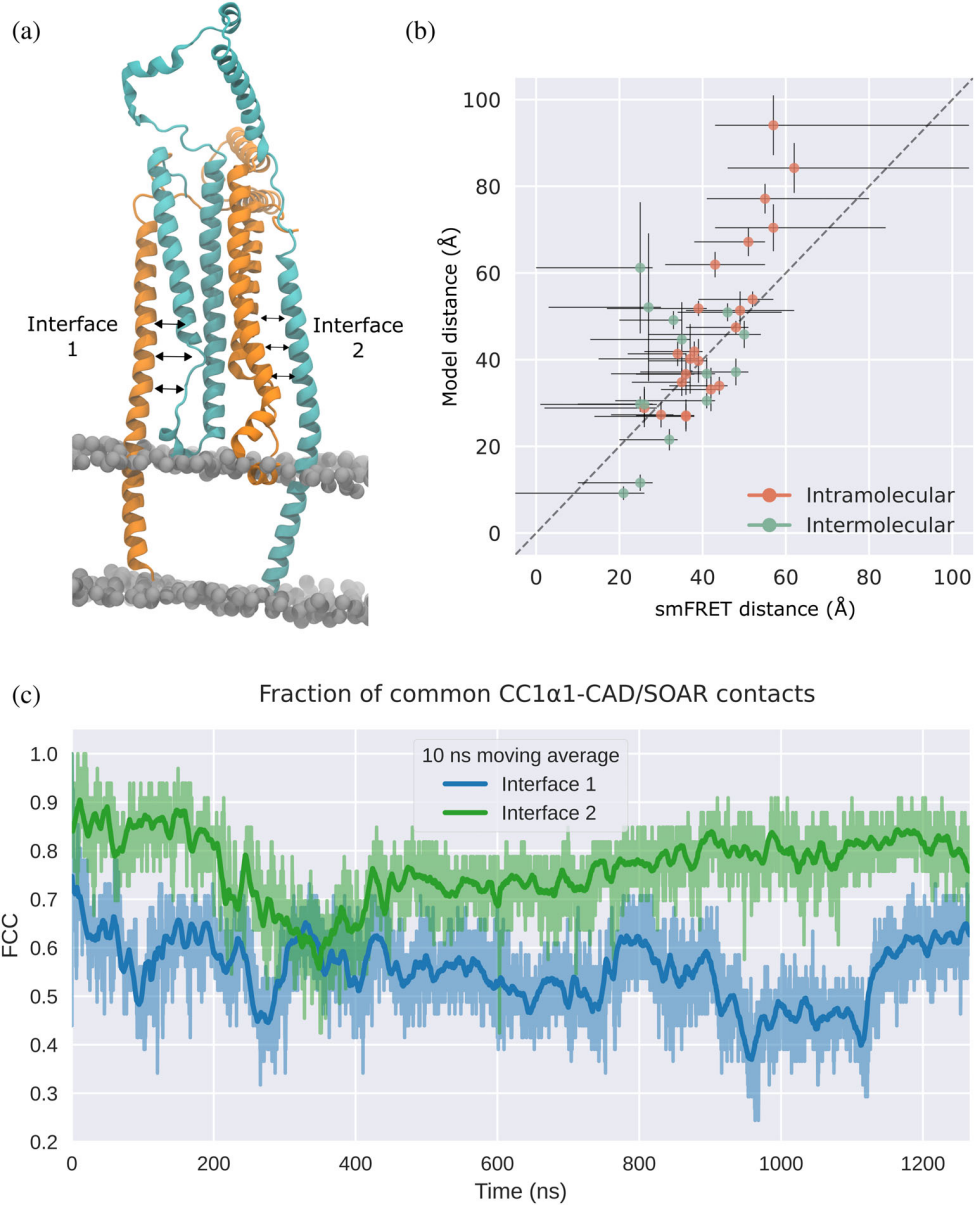
(a) Dimeric STIM1 model (residues 214–443) in domain‐swapped configuration. The two monomers are colored in cyan and orange, respectively. (b) Center of mass distances measured in our dimeric STIM1 model compared with distances derived from single‐molecule Förster resonance energy transfer (smFRET) measurements (van Dorp et al., 2021). smFRET distance error bars are set according to the distance range reported in reference (van Dorp et al., 2021), model distance error bars denote the standard deviation. Residue pairs are listed in Table S2. (c) Fraction of common contacts between CC1α1 and CAD/SOAR with respect to the monomeric CC1α1‐CAD/SOAR interface. The blue and green traces correspond to the two CC1α1‐CAD/SOAR interfaces in the dimer.

To test whether the binding interface uncovered by our simulations is compatible with a domain‐swapped configuration suggested recently by van Dorp et al, (2021) and to complement our earlier monomeric model, we constructed a membrane‐embedded dimeric STIM1 model comprising the trans‐membrane domain (residues 214–233) and two copies of our cytosolic STIM1 model (residues 234–443) in domain‐swapped configuration (see Figure 5a). The dimeric model was initially prepared using the CC1α1‐CAD/SOAR binding interface obtained from our monomeric model, following the suggestion of van Dorp et al. (2021), who assumed both bound configurations (CC1α1‐CAD/SOAR and domain‐swapped CC1α1‐CAD/SOAR′) use the same binding interface. This initial interface was then allowed to equilibrate during unrestrained MD (see Section 4.4 and File S2).

Throughout unrestrained MD simulations (visualized in Movie S2), we monitored the fraction of common contacts (FCC) between CC1α1 and CAD/SOAR with respect to the initial binding interface taken from the monomeric model. We found that after a transition period, the FCC calculated for the two interfaces in our dimer stabilized at relatively high values. One of the two domain‐swapped dimer interfaces was more reminiscent of the monomer interface and more stable, with FCC ≈ 0.8, indicating that around 80% of all CC1α1‐CAD/SOAR contacts present in our initial binding interface are preserved in the membrane‐embedded STIM1 dimer (Figure 5c). While the other interface was more dynamic, it relaxed back to the initial binding configuration even after large perturbations (FCC ≈ 0.25). When compared with our monomeric model, we found no reduction in the stability of CC1α1‐CAD/SOAR binding in the domain‐swapped dimer (Figure S10), and interface contacts were largely preserved (Figure S11).

Although the CC1α1‐CAD/SOAR interface proposed in van Dorp et al. (2021) is somewhat shifted with respect to the interface in our model (see Section 3 and Figure S12), inter‐residue distances calculated for our model are in good agreement with distances derived from smFRET measurements (see Figure 5b and Table S2; van Dorp et al., 2021). While a selection of experimentally determined distance restraints reported in van Dorp et al. (2021) was already included in the construction of our dimeric model, the agreement with a larger test‐set of 37 different smFRET‐derived distances tended to increase over the course of our unrestrained MD simulation (Figure S13).

Our simulations also agree with dynamical properties reported by van Dorp et al. (2021), such as the high mobility of helices CC1α2/α3 (Figure S14) and their transition between a compact and a splayed state (Figure S15), or the high flexibility of the CAD/SOAR apical region (Figure S14).

The dimeric STIM1 model also allowed us to study the R426L mutation in greater detail, which was experimentally found to strongly reduce CRAC channel currents (Figure S5; Fahrner et al., 2014; Ma et al., 2020; Muik et al., 2011). In our simulation of the R426L mutant, the more canonical coiled‐coil heptad repeat of R426L led to a notable increase in CC2‐CC3 interactions (Figure S16). Since a NMR spectroscopy solution structure suggests that STIM1–Orai1 binding requires CC3 to detach from CC2 (Derler et al., 2016; Stathopulos et al., 2013), enhanced CC2–CC3 interactions could serve as a potential explanation for R426L inactivity. Further research is required in this direction. Moreover, MD simulations using our dimeric model reinforced our conjecture that in the A397S mutant, weakened CC1α1‐CAD/SOAR interactions are compensated by enhanced CAD/SOAR‐membrane interactions. When compared with the WT, the CAD/SOAR apex sticks more tightly to the membrane in dimeric A397S (Figure S16).

**FIGURE 6:**
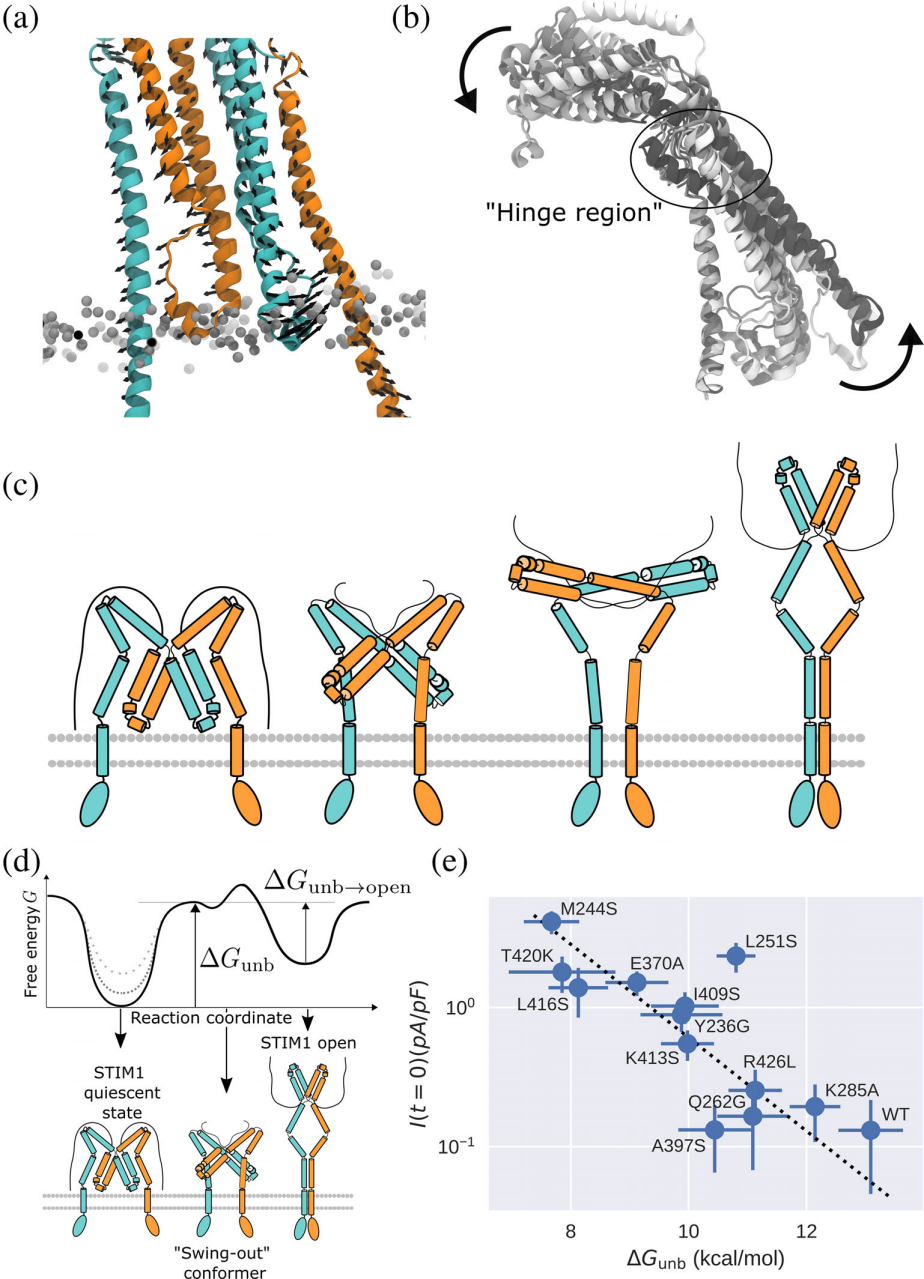
(a) Primary mode of motion as calculated via principal component analysis of backbone atom fluctuations in a STIM1 “domain‐swapped” dimer. The two monomers are colored in orange and cyan, respectively. Black arrows indicate the primary (0th) mode of motion of Cα atoms (accounting for 36% of the variance of fluctuations). (b) Tilt‐up motion during CC1α1‐CAD/SOAR unbinding metadynamics of a STIM1 monomer. From white to black, frames are overlaid with a timestep of 30 ns. (c) “Swing‐out” model of STIM1 activation. From left to right, STIM1 initially occupies its quiescent, tight state (in a putative domain‐swapped configuration). The onset of STIM1 activation involves both CAD/SOAR domains swinging outwards in opposite directions. STIM1 then elongates into a parallel dimer. (d) Hypothetical free energy profile of STIM1 opening. Different mutations in the CC1α1‐CAD/SOAR binding interface are assumed to affect only the free energy of the STIM1 quiescent state (dotted gray lines). (e) Δ*G*_unb_ and *I* (*t* = 0) are fitted according to Equation (1).

Finally, one main advantage of our STIM1 dimer is that it paints a more realistic picture of how the STIM1 active state may be accessed upon ER Ca^2+^ store depletion. To obtain a first‐order approximation of collective motion that could support a transition from the quiescent to the elongated active state, we performed a principal component analysis (PCA) of atomic fluctuations recorded during unconstrained MD simulations of our dimeric model. The leading modes of motion recovered from our PCA suggest that the two CC1α1′‐CAD/SOAR and CC1α1‐CAD/SOAR′ complexes fluctuate with opposite phase, with the two CAD/SOAR domains swinging outwards in opposite directions (Figure 6a). Similar collective motion is obtained from the lowest frequency mode of an anisotropic network model built from Cα atoms of the dimeric model (Figure S17), which indicates that this motion directly emerges from the global structure of the cytosolic STIM1 domain. While we could not experimentally test the functional importance of this finding, the observed swing‐out motion represents a plausible and intuitive mechanism for the onset of STIM1 activation that complies with previous suggestions (Jennette et al., 2022; van Dorp et al., 2021).

## 3 DISCUSSION

STIM1 is a highly dynamic protein that undergoes a large‐scale conformational transition when switching between its quiescent and active states. At the same time, this transition is finely balanced to ensure just the right activation barrier. Any disturbance of STIM1 activation leads to serious human diseases such as immunodeficiency or autoimmunity. Since the available STIM1 structures comprise only relatively small fragments of the protein, CRAC channel research has been impeded by insufficient structural information on STIM1, necessitating effortful mutation screenings, crosslinking approaches or single‐molecule FRET experiments to draw conclusions about how STIM1 is structured in its different states (Fahrner et al., 2014, 2018; Hirve et al., 2018; Ma et al., 2015, 2020; Muik et al., 2011; van Dorp et al., 2021). Previous studies have thus provided crucial information about STIM1 interaction sites, but for the most part, they only supplied rough estimates on how STIM1 is structurally organized in a given conformational state. While resolving full‐length STIM1 in an x‐ray or NMR structure has proved challenging (Cui et al., 2013; Rathner et al., 2021; Stathopulos et al., 2013; Yang et al., 2012), in silico structural modeling is an efficient alternative approach that allows integrating different experimental datasets into a comprehensive structural model. By combining all known information on the composition of C‐terminal STIM1 in the quiescent state into a refined model, we provide a base for future STIM1 research, enabling easy visualization and accurate prediction of intramolecular interactions.

In this study, our model successfully guided experimental research towards key intramolecular binding sites. By selectively destabilizing the STIM1 autoinhibitory clamp, we could identify novel critical sites controlling the STIM1 quiescent state. Binding sites predicted to be integral to the CC1α1‐CAD/SOAR clamp consistently led to enhanced STIM1 and Orai1 activation when mutated in the patch clamp experiment. Conversely, sites with low CC1α1‐CAD/SOAR binding score generally failed to strongly affect STIM1 function upon mutation. Equipped with detailed information about interactions of individual residues in the CC1α1‐CAD/SOAR binding interface, we could elucidate the molecular mechanism underlying various subtleties of STIM1 function, such as the nuanced impact of different point‐substitutions at a given position or the complex interplay of hydrophobic and electrostatic CC1α1‐CAD/SOAR interactions. By extending our model to a STIM1 dimer, we could show that the CC1α1‐CAD/SOAR binding interface predicted in our monomeric structure remains stable even in a putative domain‐swapped dimeric configuration (van Dorp et al., 2021), which lends further credibility to our monomer model predictions.

Since our model incorporates data by Ma et al. (2015) and van Dorp et al. (2021), it is worthwhile to compare our results with the binding interfaces proposed by these authors. The CC1α1‐CAD/SOAR binding interface featured in our model is similar to the one proposed in (Ma et al., 2015), but differs insofar as residues 261 and 419 are not directly opposed but moved apart slightly (Figure S12). Interestingly, although our model and the one presented in (van Dorp et al., 2021) overlap in their underlying experimental input data, the two models differ considerably. When compared with the interface proposed in Figure 5e in (van Dorp et al., 2021), in our model CAD/SOAR is shifted away from the ER membrane by about 18 Å (Figure S12). One possible reason for this discrepancy is that the docking simulations performed in (van Dorp et al., 2021) did not directly implement experimental contacts as distance restraints. Rather, the authors used a more indirect approach whereby FRET measurements were used to construct an ensemble of models (Figures 5 and fig. S1b,c in van Dorp et al., 2021) and then distance restraints were derived from contacts observed in the average structure of this ensemble. Note that, due to this shift in the position of CAD/SOAR, in our model the CAD/SOAR domain does not fully insert into the ER membrane. Rather, its apex domain just about reaches the membrane (Figure 5a; Höglinger et al., 2021).

During the preparation of this article, a study appeared which similarly investigates CC1α1‐CAD/SOAR binding by means of an alanine mutagenesis screen combined with FRET measurements (Shrestha et al., 2022). This report agrees with our own findings in that it also highlights positions M244, L265, and I409 as crucial CC1α1‐CAD/SOAR interaction sites. Our own study provides in‐depth complementary information to the results of Shrestha et al. by elaborating on the detailed interactions of each binding residue and on how these are affected by varying substitutions.

Many details of the STIM1 activation mechanism are still unknown. Prominent among them is the precise process by which CC1α1‐CC1α1′ homomerization drives CC1α1‐CAD/SOAR unbinding, initiating STIM1 elongation and the dimerization of all three CC1 subdomains (van Dorp et al., 2021). Our models shed some light on this puzzle, since they highlight the mechanism of CC1α1‐CAD/SOAR detachment and allow a direct analysis of the collective dynamics of dimeric STIM1. As for CC1α1‐CAD/SOAR detachment, our metadynamics simulations suggest this transition resembles a tilt‐up motion, where CAD/SOAR swings out and away from the membrane plane, while the CC1α1,2 hairpin tilts in the opposite direction (Figure 6b). CC1α1‐CAD/SOAR contacts between the C‐terminal end of CC1α1 and the “base” of CAD/SOAR (i.e., residues 260–266 and 416–427) act as a pivot for this tilt‐up motion, resulting in prominently high binding scores *S*_*i*_ for residues in this region. In line with this interpretation, lysine substitions of T420 and Q262, which are both situated in this hinge region (Figure S2a), lead to constitutive STIM1 activation (Figure S6).

Activation of Orai1 requires the STIM1 apex to point away from the ER membrane, hence it must undergo a ≈ 180° rotation. The collective swing‐out motion observed in our simulations of a STIM1 dimer could provide a valuable hint as to how STIM1 overcomes the autoinhibitory CC1α1‐CAD/SOAR clamp and transitions towards a fully dimerized elongated conformation (Figure 6a). This collective motion, together with the tilt‐up movement observed in our monomeric model, suggests a “swing‐out” activation mechanism by which the two CAD/SOAR domains tilt sideways (away from the plane connecting the two CC1α1 helices), with the C‐terminal end of CC1α1 and the “base” of CAD/SOAR acting as a pivot.

Since our simulations allow probing the stability of the STIM1 quiescent state for different mutants and our patch clamp measurements report on the resulting fraction of pre‐activated Orai1 channels, we can draw conclusions about STIM1–Orai1 binding for different CC1α1‐CAD/SOAR binding strengths. A simple theoretical model allows us to formally relate our calculated Δ*G*_unb_ and the experimental *I* (*t* = 0). If we assume that mutations in the CC1α1‐CAD/SOAR binding interface only affect the free energy of the STIM1 quiescent state (Figure 6d), we can relate Δ*G*_unb_ and *I* (*t* = 0) as

(1) $$I_{0}=c_{0}\cdot {\left(\frac{1}{1+c_{1}e^{\Delta G_{\mathrm{unb}}/\mathrm{RT}}}\right)}^{n}$$

where *c*_0_ and *c*_1_ are constants and *n* denotes the cooperativity parameter (see Appendix S1 for details). Via the constant *c*_1_, we can not only probe the free energy difference between the STIM1 closed and CAD/SOAR‐detached states, Δ*G*_unb_, but also the free energy difference between the CAD/SOAR‐detached state and the fully open STIM1 state, $\Delta G_{\mathrm{unb}\to \text{open}}=\Delta G_{\text{quiesc}\to \text{open}}-\Delta G_{\mathrm{unb}}$ (Figure 6d). Relating Δ*G*_unb_ and *I*(*t* = 0) according to this model yields $\Delta G_{\mathrm{unb}\to \text{open}}=-4.81\pm 0.01$ kcal/mol, which is similar to the unbinding free energy Δ*G*_unb_ calculated for mutants such as M244S. For the cooperativity parameter, we obtain *n* = 0.46 ± 0.15 (Figure 6e). In accordance with previous findings (Hoover & Lewis, 2011), our combined metadynamics and patch clamp results thus indicate anti‐cooperative STIM1–Orai1 binding, that is, the binding of one STIM1 dimer to Orai1 interferes with the binding of subsequent STIM1 proteins (Bisswanger, 2008; Price et al., 2001). The overall good validity of Equation (1), shown in Figure 6e, demonstrates that CC1α1‐CAD/SOAR unbinding acts as the crucial parameter in the signal cascade conducive to CRAC channel opening. In particular, for the different mutants considered here, changes in CC1α1‐CAD/SOAR binding prevail over the many other factors, for example, STIM1 oligomerization or Orai1 gating, which are not captured by our simulations.

These additional influencing factors cannot at present be tackled based on our models alone. Uncovering the full STIM1 activation mechanism at atomistic detail thus continues to pose a formidable challenge. Still, the insights provided by our models act as steppingstone for future CRAC channel research, rendering accessible the different stages of the STIM1 activation cascade with ever‐increasing detail.

## 4 MATERIALS AND METHODS

### 4.1 Model construction

After restoring the WT sequence of the crystallized CAD/SOAR domain (PDB id 3TEQ; Yang et al., 2012), CC1α1 (PDB id 6YEL; Rathner et al., 2021) was docked to CAD/SOAR using the haddock 2.4 webserver (De Vries et al., 2010; van Zundert et al., 2016). As active residues, we selected residues 258, 261, 416, 419, and 423 (Ma et al., 2015). Unambiguous restraints were added to reinforce pairing between bissulfosuccinimidyl suberate (BS^3^) crosslinked residues (see van Dorp et al., 2021; Figure S5c). The best‐scoring structure (Figure S18a) was complemented by extracting helices CC1α2,3 from the CC1 NMR model (PDB id 6YEL) and joining them to the docked CC1α1‐CAD/SOAR fragments. For further details, see Methods S1.

### 4.2 Simulation methods

The resultant structure was first energy minimized and equilibrated in implicit solvent for 100 ps at 200 K. The model was then solvated using explicit TIP3P water and neutralized by adding counterions. This was followed by energy minimization, heating to 300 K, and a short (10 ns) equilibration run. Protonation states of titratable residues were determined using protein electrostatics calculations with tapbs (Kieseritzky & Knapp, 2008) and karlsberg 2.0 (Rabenstein & Knapp, 2001). With the correct protonation states, we repeated the procedure of solvating, neutralization, heating, and equilibration. This final setup was thoroughly equilibrated over 500 ns. All MD simulations were performed with namd 2.14 (Phillips et al., 2020) and namd 3.0 alpha (NAMD 3.0, 2022), and the charmm36m force field (Huang et al., 2016). The NpT ensemble was employed with a constant temperature of 300 K using Langevin dynamics. Pressure was set to 1 atm with the Langevin piston method. Long‐range electrostatic interactions were handled with the particle‐mesh Ewald method (Essmann et al., 1995). Dynamics were calculated using the velocity Verlet algorithm with an integration time step of 2 fs. H‐bonds were restrained using ShakeH.

All trajectory and free energy analysis was done using custom python code employing the mdtraj (McGibbon et al., 2015), pytraj (Nguyen et al., 2015; Roe & Cheatham, 2013), ProDy (Zhang et al., 2021), and gRINN (Serçinoǧlu & Ozbek, 2018) packages.

### 4.3 Metadynamics methods

For our well‐tempered metadynamics simulations (Barducci et al., 2008) we chose two collective variables: (1) the center of mass distance between CAD/SOAR and the “base” of CC1α1 (based on Cα positions of residues 360–425 and 240–243, respectively), and (2) the number of contacts between CAD/SOAR (using residues 350–435) and CC1α1 (using residues 235–270). Gaussian hills with an initial height 0.3 kcal/mol and a bias temperature of 3300 K were added every 1000 steps using colvars (Fiorin et al., 2013). The hill width in the distance and contact dimensions were set to 0.125 and 0.25 Å, respectively.

All simulations were propagated up to a specific cutoff time, which was determined by calculating the number of contacts, *N*_*c*_, between CC1α1 and CAD/SOAR and terminating the run once *N*_*c*_ fully zeroes out for at least 200 ps. To check the consistency of our results and to allow for the sampling of several different unfolding pathways, we conducted at least seven independent metadynamics runs for each mutant (Dama et al., 2015; Laio & Gervasio, 2008; Pietrucci, 2017). Two‐dimensional free energy surfaces were obtained by integrating out the contact collective variable.

Reweighting of non‐biased observables was done using the balanced exponential reweighting scheme by Schäfer & Settanni (2020), providing weights $w_{t}^{\mathrm{bex}}$ (see Methods S1). Correct reweighting was checked by comparing the FES as calculated directly via the metadynamics bias potential with the FES calculated via the reweighted histogram of the biased collective variables. In all cases, this yielded excellent agreement (Figure S19). The biased contact frequency $\omega_{\mathrm{ij}}^{b}$ was calculated (using Contact Map Explorer; Swenson & Roet, 2020) directly from our metadynamics runs for a pair of binding residues *i* and *j*, one of which is in CC1α1 and one in CAD/SOAR. The reweighted contact frequency *ω*_*ij*_ was obtained by reweighting $\omega_{\mathrm{ij}}^{b}$ with weights $w_{t}^{\mathrm{bex}}$. Based on the reweighted contact frequency, we define a binding score $S_{i}=\sum_{j}\omega_{\mathrm{ij}}$ to describe the binding of residues *i*, *j* in opposing protein domains. By construction, this score reflects how many cross‐domain contacts a given residue forms, and how stable these contacts are throughout the detachment simulation.

### 4.4 Dimeric model

We augmented the monomeric model with the STIM1 trans‐membrane domain (residues 214–233), which was modeled as an alpha helix based on its primary sequence using modeller (Webb & Sali, 2016). The extended model, comprising residues 214–443, was dimerized using haddock. From among the output clusters with the correct relative orientation, the top‐scoring structure was selected (Figure S18b). By construction, the resultant dimer did not manifest a domain‐swapped configuration. To model this conformation, the loops connecting CC1α2 to the base of CAD/SOAR were manually shifted to connect to the CAD/SOAR domain of the respective opposite monomer while keeping all other atomic positions fixed.

After assigning protonation states (see above), the model was embedded in a membrane using CHARMM GUI (Jo et al., 2008). The membrane patch consisted of DDPC, DLPE, and DMPI with a lipid ratio of 4:2:1 (Van Meer et al., 2008) and a side length of 131 Å. The system was neutralized, heated, and equilibrated as described in Section 4.2. After an equilibration period of 300 ns, conformational sampling was performed in three independent 500 ns replicas. For the FCC study shown in Figure 5, one replica was extended to around 1.2 μs. Inter‐residue distances for residue pairs listed in Table S2 were calculated for the final 500 ns of three independent runs of unrestrained MD (Figure 5). smFRET‐derived distances were calculated based on peak FRET values reported in reference (van Dorp et al., 2021) and applying standard Förster theory, $d^{\exp}=R_{0}{\left(\left(1/E\right)-1\right)}^{1/6}$, with *R*_0_ = 5.1 nm (Stryer & Haugland, 1967), and subtracting 1 nm to account for fluorophore linker length. The PCA of atomic fluctuations was performed for Cα atoms over the final 500 ns of all three replicas after aligning of coordinates. The anisotropic network model was constructed with ProDy (Zhang et al., 2021).

### 4.5 Molecular cloning and mutagenesis

As highlighted by our analysis, different point substitutions can elicit markedly different effects on STIM1 function. Accordingly, the substitutions used in our mutagenesis studies were tailored on each specific position, considering their surroundings as described by our model. Aiming to disrupt hydrophobic CC1α1‐CAD/SOAR interactions, hydrophobic amino acids were mutated to serine (M244S, M245S, V396S, and I409S). To neutralize electrostatic interactions, charged amino acids were mutated to hydrophobic alanine (K267A, E270A, K366A, E373K, E381A, K384A, E370A, K377A, and K413A). In specific cases, we suspected that alanine substitutions could significantly enhance CC1α1‐CAD/SOAR hydrophobic interactions and thus substituted charged amino acids by serine or by amino acids with opposite charge (E263S, K366S, E370K, K377S, K413S, and E373K). To disturb interactions involving polar residues, we opted to either remove sidechains entirely via glycine substitutions or introduce a long, charged lysine sidechain (Q262K, Q262G, T420K, T420G, Y236G, and Y236K).

N‐terminally pECFP‐labeled and pEYFP‐labeled human STIM1 (accession number NM_003156) were made available by T Meyer (Stanford University). N‐terminal pECFP‐STIM1 mutants and N‐terminal pEYFP‐STIM1 mutants were built with the aid of the QuikChange XL site‐directed mutagenesis kit (Stratagene, California, USA). For the generation of double‐tagged STIM1–OASF constructs, CFP was introduced into pEYFP‐C2 via SacII and Xba1 and the OASF fragment (aa233‐474) of STIM1 was inserted via restriction sites EcoRI and SacII. Double‐tagged YFP‐OASF‐CFP mutants were built with the aid of the QuikChange XL site‐directed mutagenesis kit (Stratagene). All constructs were verified by sequence analysis.

### 4.6 Cell culture and transfection

Human embryonic kidney 293 (HEK293, provided by DSMZ – German Collection of Micro‐organisms and Cell Cultures GmbH) cells were cultured in DMEM supplemented with 2 mM 1‐glutamine, 100 μg/mL streptomycin, 100 U/mL penicillin and 10% fetal calf serum. Cells were grown at 37°C in a 90% humidity‐controlled and 5% CO_2_‐controlled incubator. TransFectin™ lipid reagent (Bio‐Rad) was used to perform transient transfection as previously shown in reference (Derler et al., 2006). The plasmid amounts used were 1 μg STIM1 WT/mutants:1 μg Orai1 WT in electrophysiological experiments, while in our FRET studies a ratio of 1:1 μg for CFP‐STIM1:YFP‐STIM1 was used for intermolecular homomerization experiments and 0.7 μg YFP‐OASF‐CFP were used for intramolecular OASF sensor experiments. All experiments were performed 24 h after transfection. HEK293 cells were routinely checked for mycoplasma contamination using Venor GeM Advanced Mycoplasma Detection Kit (Minerva Biolabs GmbH).

### 4.7 FRET microscopy

Confocal FRET microscopy of HEK293 cells was carried out at room temperature. For the experimental setup, a CSU‐X1 Real‐Time Confocal System (Yokogawa Electric Corporation, Japan) fitted with two CoolSNAP HQ2 CCD cameras (Photometrics, Arizona, USA) was used. Additionally, a dual port adapter (dichroic: 505lp, cyan emission filter: 470/24, yellow emission filter: 535/30, Chroma Technology Corporation, Vermont, USA) was part of the installation. This configuration was connected to an Axio Observer.Z1‐inverted microscope (Carl Zeiss, Oberkochen, Germany) with two diode lasers (445 and 515 nm, Visitron Systems, Puchheim, Germany). All components were placed on a Vision IsoStation anti‐vibration table (Newport Corporation, California, USA). The VisiView software package (v2.1.4, Visitron Systems) was employed for controlling the confocal system and image recording. Due to cross‐excitation and spectral bleed‐through, it was necessary to perform image correction before any kind of FRET calculation. Therefore, cross‐excitation calibration factors were experimentally determined for all expressed DNA constructs on each day that measurements were performed. Apparent FRET efficiency *E*_app_ was calculated using code (Derler et al., 2006) employing MATLAB (v7.11.0, The MathWorks, Inc., Massachusetts, USA; The MathWorks Inc, 2015) and implementing the method described in (Zal & Gascoigne, 2004).

### 4.8 Electrophysiology

Patch clamp electrophysiology recordings of HEK293 cells were carried out at room temperature. The whole‐cell configuration was exclusively used in all experiments with two Ag/AgCl electrodes serving as both recording as well as reference electrodes. A 1 s voltage ramp covering the range of −90 to +90 mV was applied every 5 s; the holding potential was thereby set to 0 mV. The current amplitudes recorded at −74 mV during every voltage ramp were used for data evaluation. Passive store depletion was initiated by the internal pipette solution containing (in mM): 145 Cs methane sulfonate, 20 EGTA, 10 HEPES, 8 NaCl, and 5 MgCl_2_ at pH 7.2. The standard extracellular solution contained (in mM) 145 NaCl, 10 HEPES, 10 CaCl_2_, 10 glucose, 5 CsCl, and 1 MgCl_2_ at pH 7.4. All experiments were carried out on at least two different days. Leak correction was applied to all recordings.

### 4.9 Statistical analysis

All experimental results are presented as mean ± SEM calculated for the indicated number *n* of experiments. For statistical comparisons, the Kolmogorov–Smirnov test was first applied to verify that the respective datasets were drawn from normally distributed populations. Levene's test was then used to test for variance homogeneity. If variance homogeneity was fulfilled, one‐way analysis of variance (ANOVA) was performed followed by Fisher's least significant difference post hoc test. Otherwise, Welch's ANOVA together with Games–Howell post hoc test was performed instead. The Grubbs test was used to eliminate outliers. The Mann–Whitney test was used for pairwise comparison to WT. The statistical significance level was set to α = 0.05 with *p‐*values ≤ 0.05 considered statistically significant.

## AUTHOR CONTRIBUTIONS

**Ferdinand Horvath:** Conceptualization (equal); formal analysis (equal); investigation (equal); methodology (equal); writing – original draft (lead). **Sascha Berlansky:** Investigation (equal); writing – original draft (equal). **Lena Maltan:** Investigation (equal); writing – original draft (equal). **Herwig Grabmayr:** Investigation (equal); writing – original draft (equal). **Marc Fahrner:** Conceptualization (equal); investigation (equal); supervision (equal). **Isabella Derler:** Conceptualization (equal); supervision (equal). **Christoph Romanin:** Conceptualization (equal); supervision (equal). **Thomas Renger:** Conceptualization (equal); supervision (equal). **Heinrich Krobath:** Conceptualization (equal); supervision (equal).

## FUNDING INFORMATION

Ferdinand Horvath and Herwig Grabmayr hold PhD scholarships of the Austrian Science Fund (FWF) PhD program W1250 NanoCell. Lena Maltan holds a PhD scholarship of Upper Austria within the FWF W1250‐B20 Upper Austria DK NanoCell Project. Additional funding was provided by Austrian Science Fund (FWF) projects P32947 (to Marc Fahrner), P30567, P32851, and P35900 (to Isabella Derler) as well as P34884 and P32778 (to Christoph Romanin).

## CONFLICT OF INTEREST STATEMENT

The authors declare no conflict of interest.

## Supporting information


**Appendix S1:** Supplementary information

**Appendix S2:** Supplementary information

**Appendix S3:** Supplementary information

**Appendix S4:** Supplementary information

**Appendix S5:** Supplementary information

## Data Availability

The data that support the findings of this study are available from the corresponding author upon reasonable request.
