# Supplementary material for: Swing‐out opening of stromal interaction molecule 1
Source: Protein Sci. 2023 Feb 15;32(3):e4571. doi: 10.1002/pro.4571 (PMC9929737; doi:10.1002/pro.4571)
Supplement: Supplementary file 2 — Appendix S2: Supplementary information [file PRO-32-e4571-s004.pdf]

Supplementary data

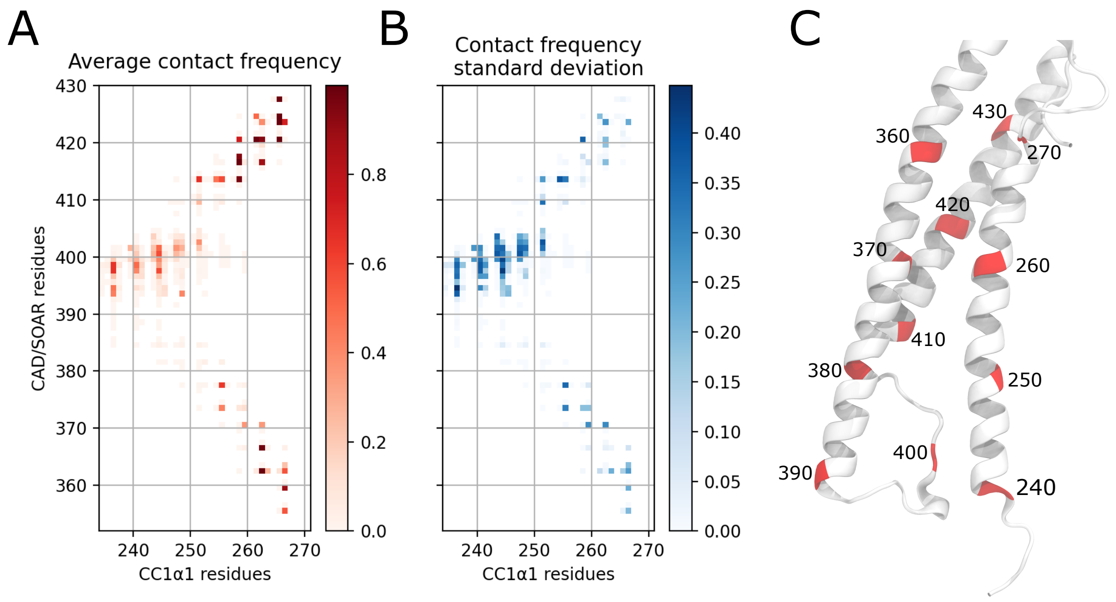

Figure S1: CC1α1-CAD/SOAR contact map for the WT model. Contact frequency average (A) and standard deviation (B) were calculated over 12 independent metadynamics runs. (C) STIM1 model with highlighted residues corresponding to the grid interval in panels (A) and (B).

#	Residue 1	Residue 2	$\langle \omega_{ij} \rangle$		#	Residue 1	Residue 2	$\langle \omega_{ij} \rangle$
1	LEU423	LEU265	1.000		26	ARG424	LEU261	0.501
2	THR420	LEU265	1.000		27	HIS398	HIS240	0.497
3	ARG424	LEU265	0.998		28	PHE394	TYR236	0.472
4	LEU416	LEU258	0.996		29	LYS413	ALA254	0.470
5	THR420	LEU261	0.996		30	THR393	TYR236	0.436
6	TYR362	GLN262	0.992		31	LEU373	GLU255	0.428
7	LEU427	LEU265	0.989		32	THR393	LEU248	0.412
8	LYS366	GLN262	0.979		33	ALA397	HIS240	0.411
9	LYS413	LEU258	0.963		34	SER399	MET244	0.408
11	THR420	GLN262	0.934		36	LEU402	LEU251	0.386
12	VAL359	HIS266	0.892		37	SER401	ASP247	0.363
13	LEU416	GLN262	0.882		38	GLU370	HIS259	0.361
14	THR420	LEU258	0.736		39	GLU370	GLN262	0.355
15	LEU423	HIS266	0.723		40	HIS398	MET244	0.330
16	LYS413	LEU251	0.663		41	SER401	LEU248	0.282
17	ALA397	TYR236	0.656		42	SER399	HIS240	0.276
18	HIS398	TYR236	0.648		43	SER401	GLU239	0.271
19	LYS377	GLU255	0.636		44	SER400	LYS243	0.269
20	ALA397	MET244	0.583		45	SER400	ASP247	0.257
21	LYS413	GLU255	0.580		46	ILE409	LEU251	0.256
22	SER400	MET244	0.578		47	TYR362	GLU263	0.256
23	TYR362	HIS266	0.558		48	VAL396	MET244	0.236
24	HIS355	HIS266	0.558		49	ASN363	HIS266	0.227
25	SER401	MET244	0.503		50	SER401	LYS243	0.221

Table S1: CC1α1-CAD/SOAR interface residues sorted by mean reweighted contact frequency $\langle \omega_{ij} \rangle$. $\langle \cdot \rangle$ refers to the average taken over 12 independent metadynamics runs.

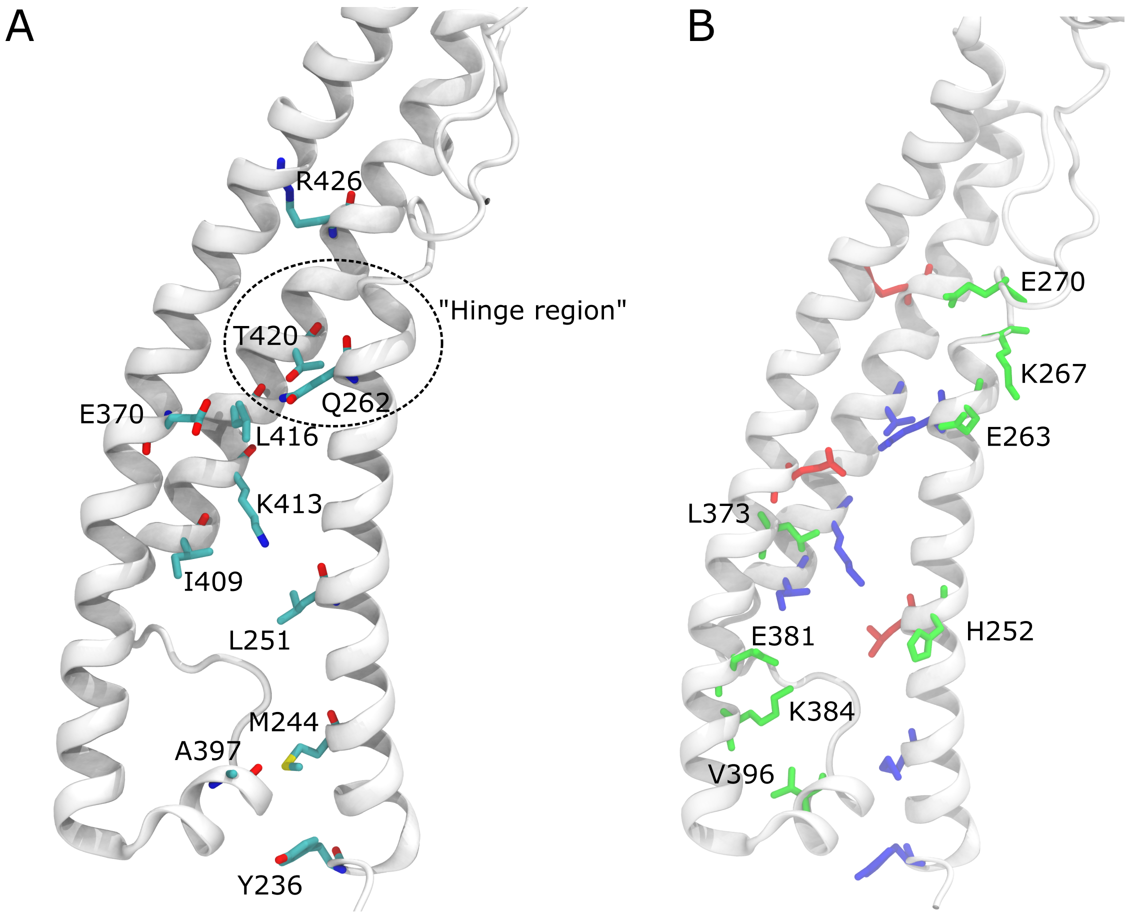

Figure S2: (A) In-silico mutated positions are shown in licorice representation in the STIM1 quiescent state model. (B) Mutated positions distinguished by method of investigation. For positions highlighted in blue, we carried out mutagenesis studies using MD simulations, patch clamp and FRET experiments. Positions marked in red were studied using MD simulations and patch clamp experiments. Positions shown in green were only studied using patch clamp experiments. Since these do not feature in panel A, they are additionally labelled.

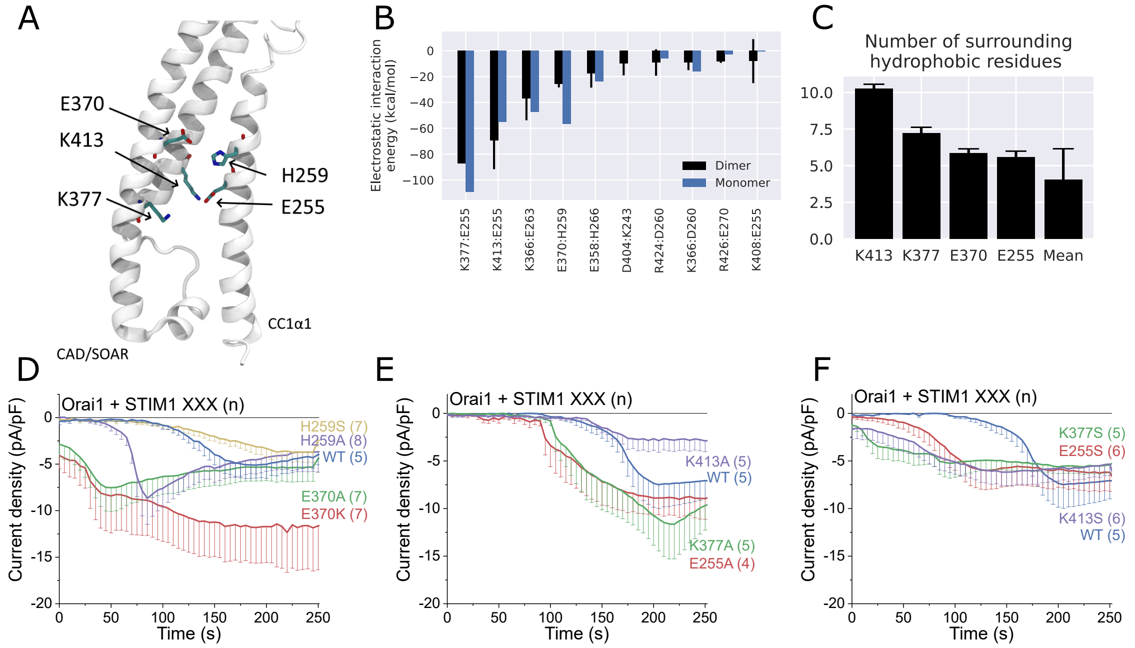

Figure S3: (A) Zoom-in on crucial interaction partners forming CC1α1-CAD/SOAR salt bridges. (B) Mean electrostatic interaction energy for the 10 strongest CC1α1-CAD/SOAR salt bridges in the STIM1 monomer and dimer, respectively. For the monomer, interaction energies were calculated over 500 ns of conventional MD. For the dimer, mean interaction energies were calculated for three 500 ns replicas. Here, error bars denote the standard deviation over the individual replicas. (C) Number of hydrophobic residues near E255, E370, K377 and K413, and the average number of hydrophobic neighbours for all charged residues in our model. Note the above-average number of hydrophobic residues surrounding K377 and K413, which suggest that mutations K377A and K413A effectively trade electrostatic CC1α1-CAD/SOAR interactions for hydrophobic ones, overall preserving STIM1 function. The number of hydrophobic neighbours was calculated using a distance cutoff of 9 Å. Distances were calculated between the Cβ atom of the charged residue and all heavy sidechain atoms of hydrophobic residues. (D-F) Patch clamp time series of whole-cell inward currents for the STIM1 WT together with ten different mutants, H259A/S, E370A/K, K377A/S K413A/S and E255A/S. Positions 377 and 413, which are surrounded by a large number of nearby hydrophobic residues (C), induce constitutive STIM1 activation when mutated to serine, but preserve store-operated STIM1 function when mutated to alanine.

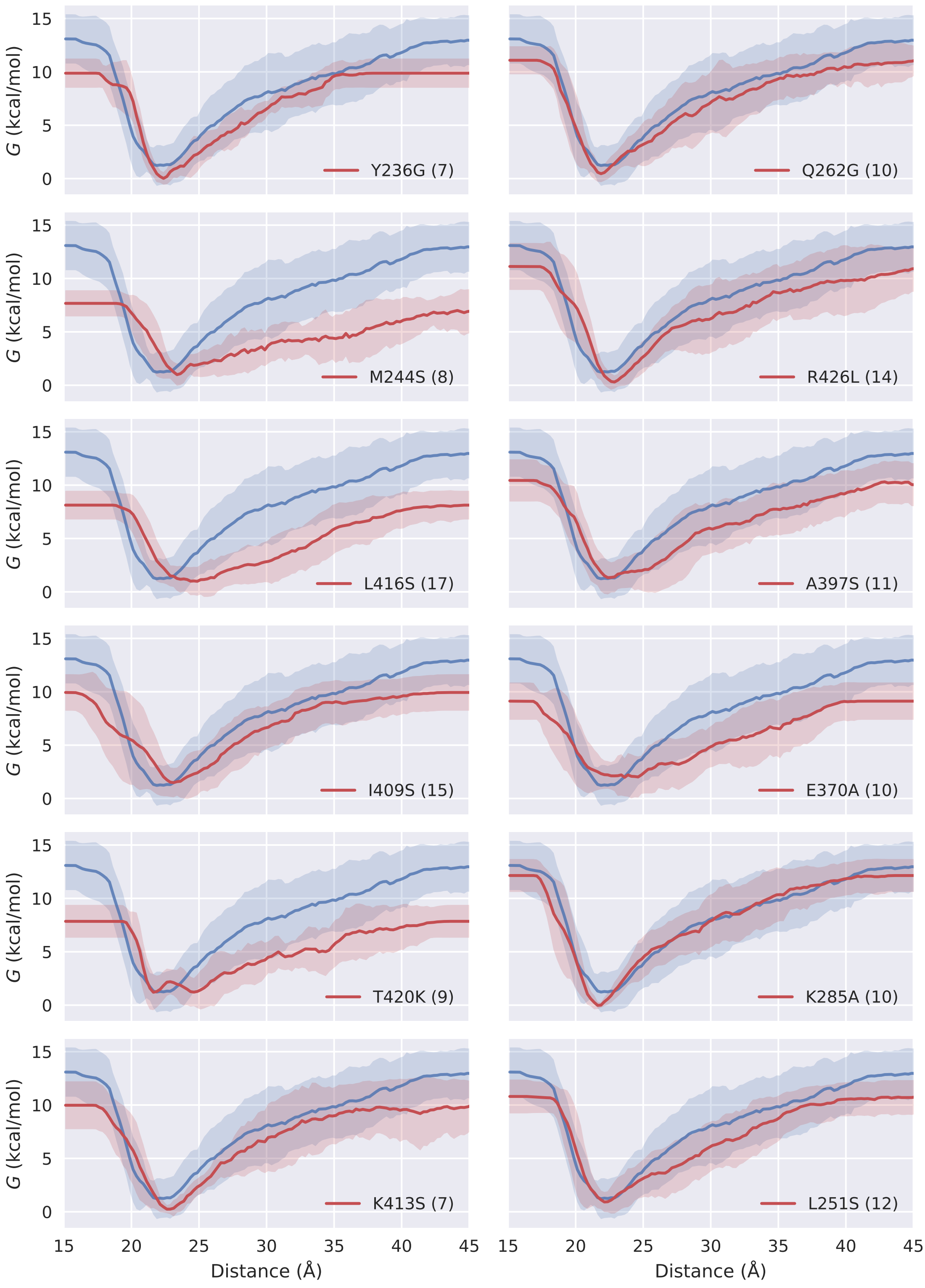

Figure S4: Free energy profiles (FEPs) of CC1α1-CAD/SOAR unbinding ΔG_{unb} as a function of CC1α1-CAD/SOAR distance for all investigated STIM1 variants. For reference, in each panel the WT FEP is shown in blue. Bracketed numbers indicate the number of independent metadynamics runs. For the WT, 12 metadynamics runs were performed. FEPs were obtained from free energy surfaces as calculated via the deposited metadynamics bias by integrating out the number of CC1α1-CAD/SOAR contacts.

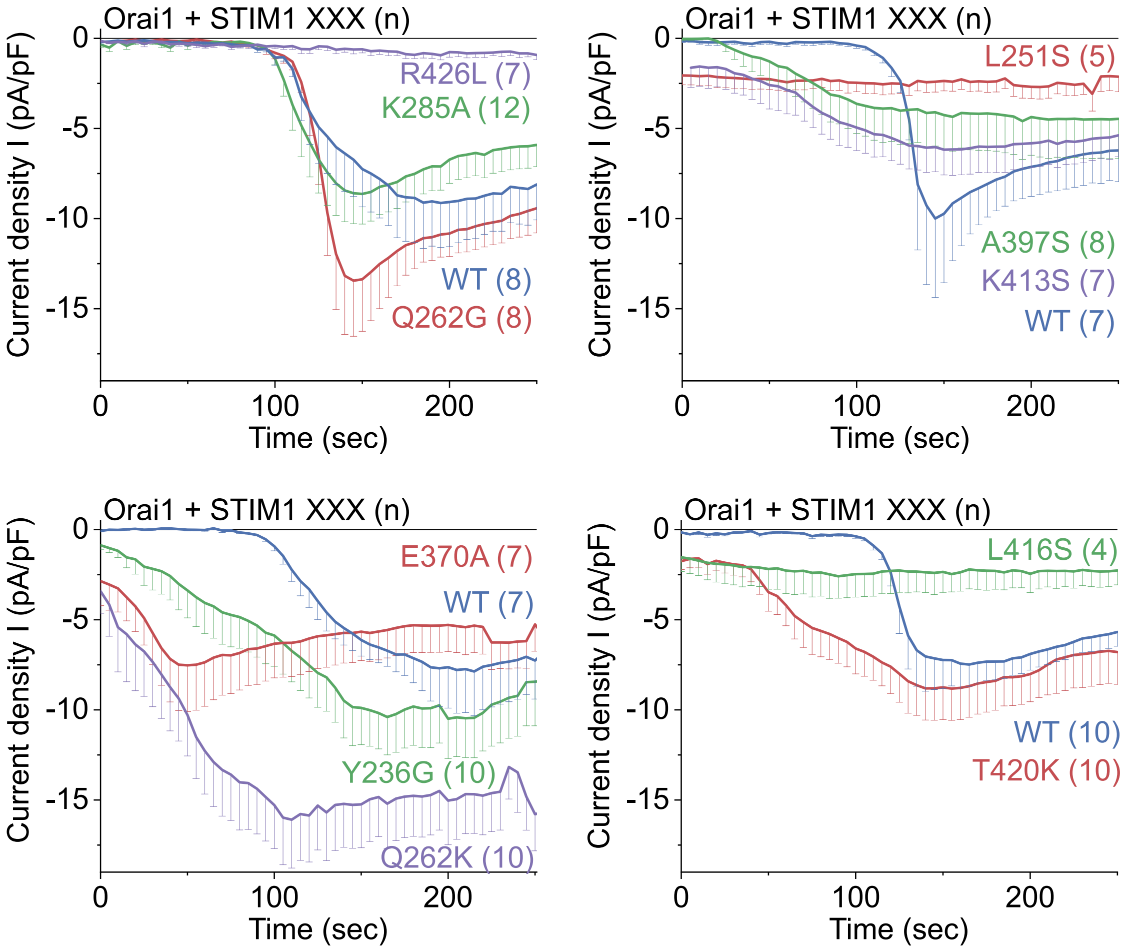

Figure S5: Time course of whole-cell inward current densities I activated by passive store depletion of HEK293 cells co-expressing Orai1 WT together with STIM1 WT or different STIM1 mutants. Error bars indicate the SEM over repeated experiments (bracketed numbers). Experiments were replicated on at least two different days using independent transfections.

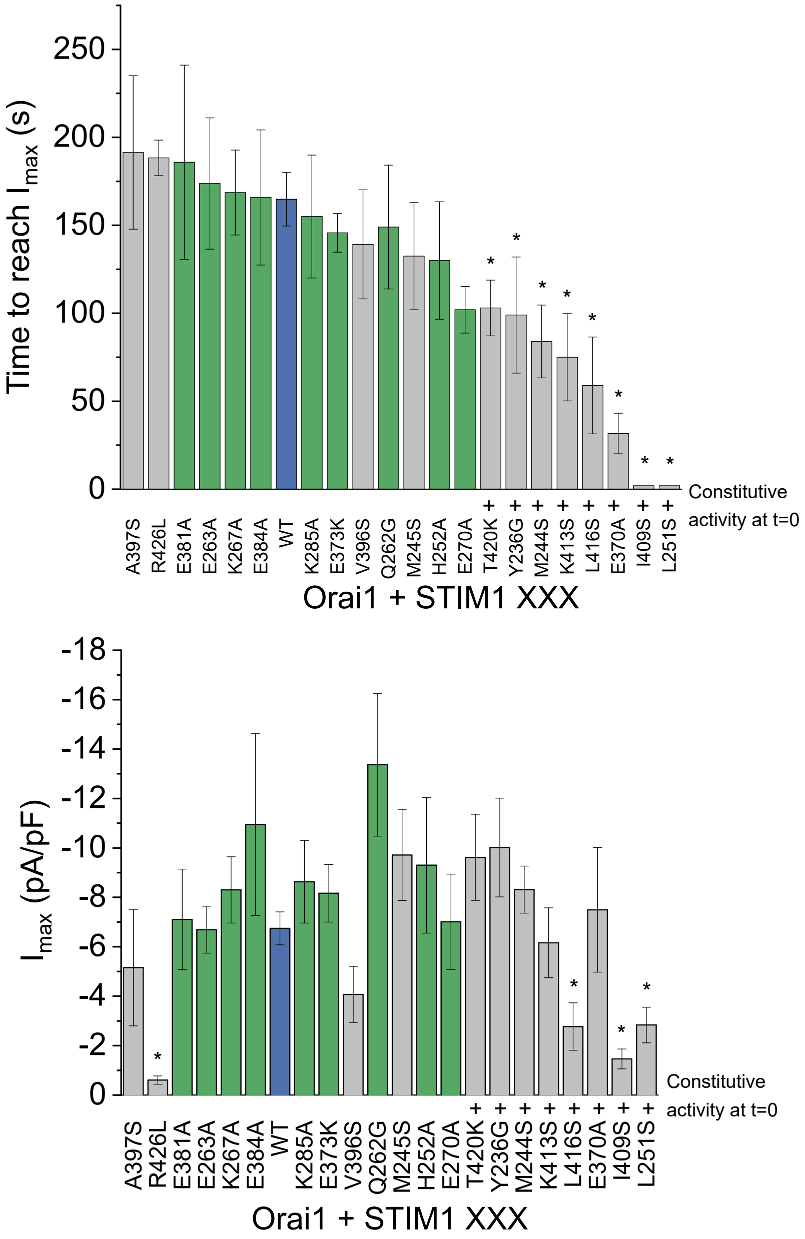

Figure S6: Overview of CRAC channel hallmarks, i.e. activation time and maximum current density $I_{\max}$, for all mutants tested in patch clamp experiments. Mutants that preserve store-operated STIM1 function are marked in green. Note that in line with their *in*ability to strongly affect STIM1 function in the patch clamp experiment, all the corresponding mutated positions exhibit a *low* simulated binding score S_i ranging from 0.01 to 0.62. Mutants marked with a "+" are constitutively active at time $t = 0$.

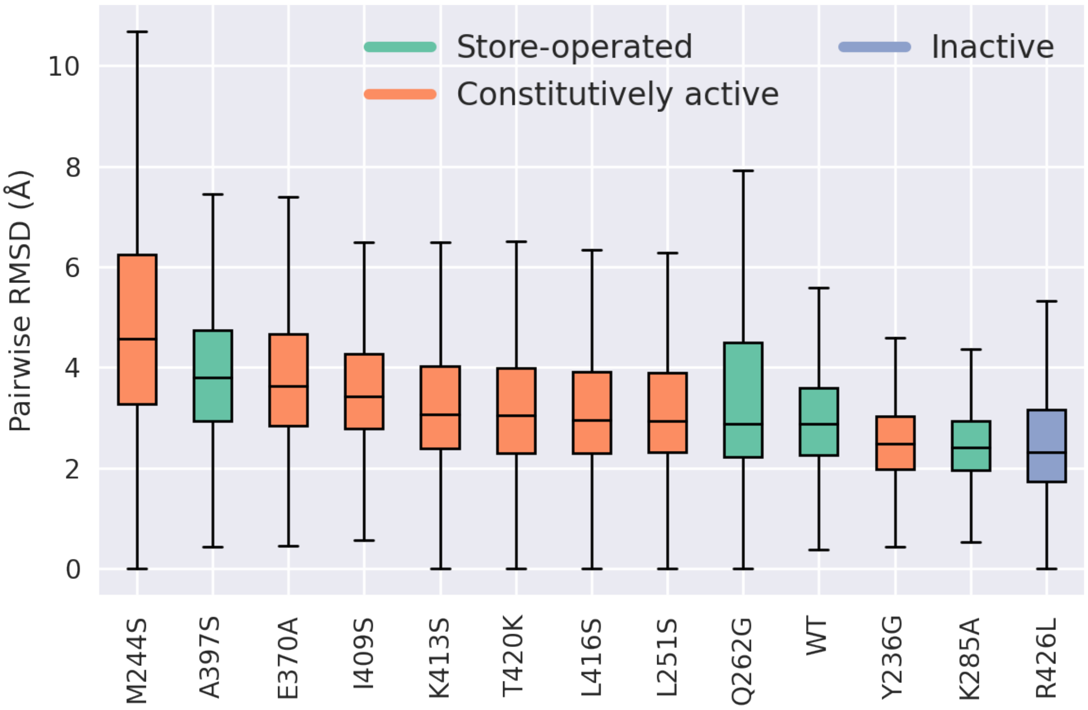

Figure S7: Pairwise RMSD between structures from the bound state ensemble (defined as $G < 2$ kcal/mol). Overall, constitutively active STIM1 mutants (orange) show a broadened bound-state free energy minimum (Figure S4). For these mutants, pairwise RMSD calculated for all bound-state conformations is on average higher than for the WT, i.e. a more diverse ensemble of conformations is accessible in the bound state. To faithfully reflect structural diversity of different CC1α1-CAD/SOAR bound states, RMSD was calculated based on Cα positions of interface residues 234-270 and 343-440.

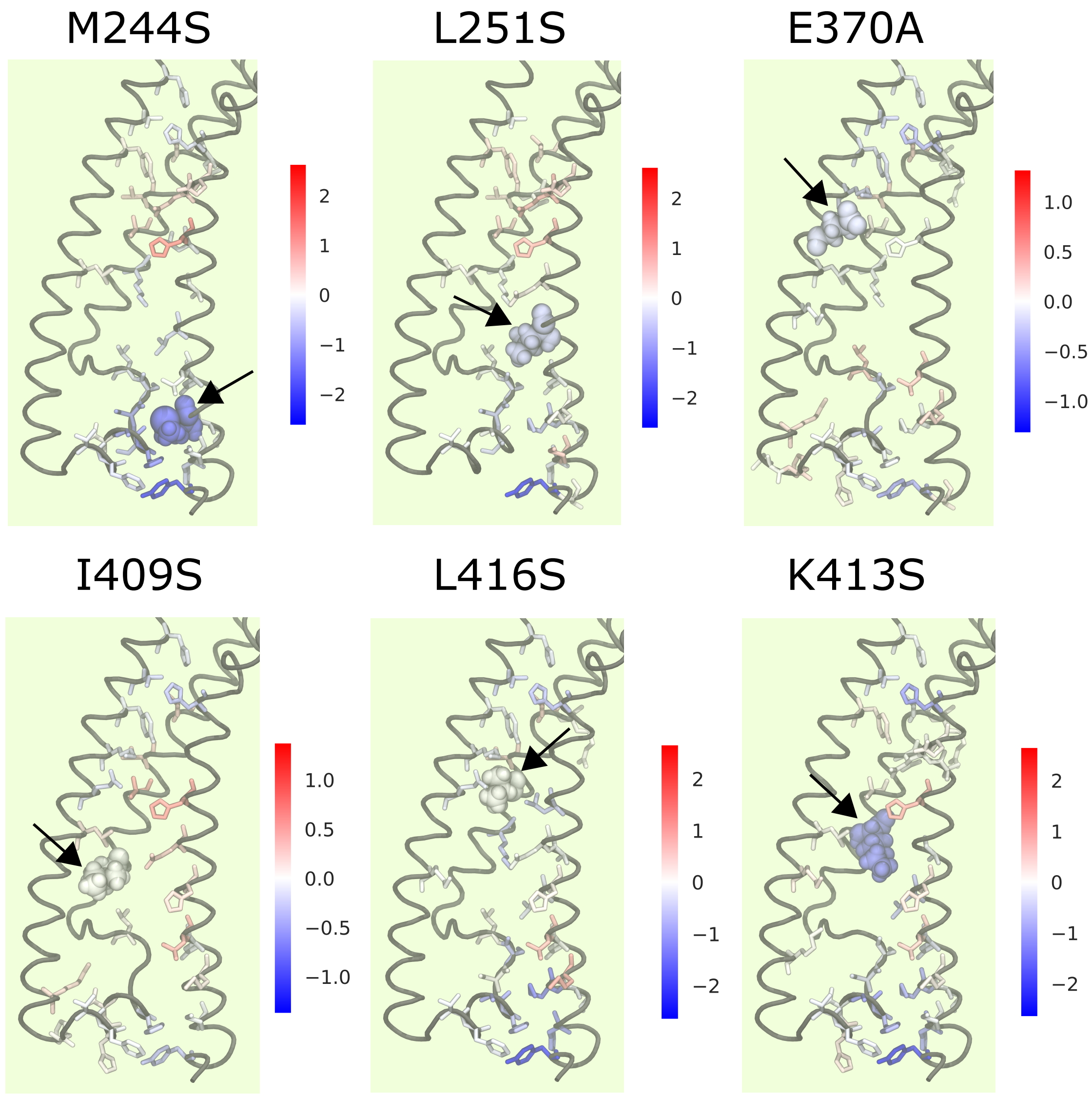

Figure S8: For selected mutants, the difference in binding score S_i with respect to the WT is color-coded onto each binding residue. Red (blue) colors indicate stronger (weaker) binding compared to the WT. For each mutant, the mutated position is highlighted using space-filling representation (arrows).

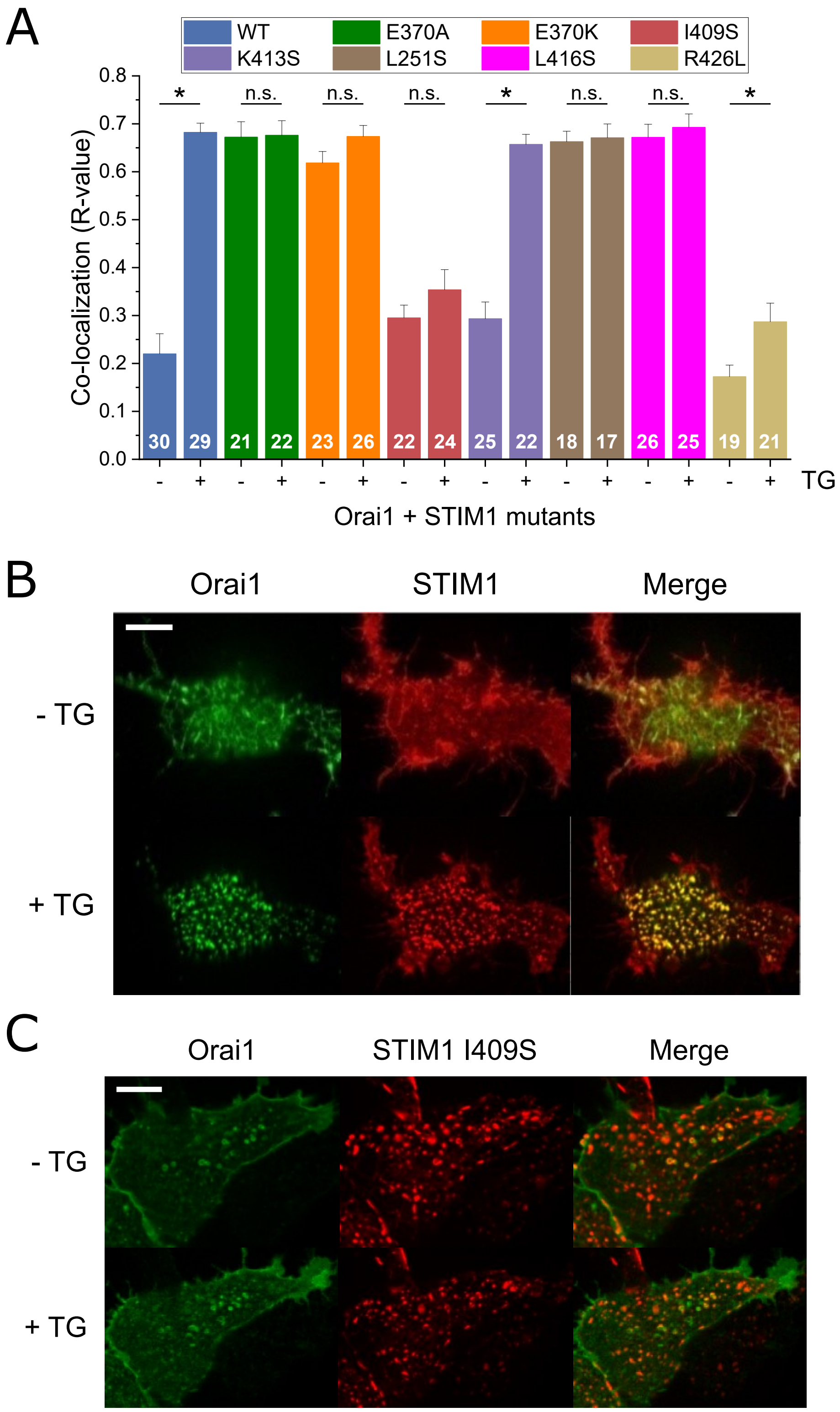

Figure S9: (A) Pearson correlation coefficient (R value) as a quantitative measurement of co-localization between the indicated YFP-STIM1 mutants and CFP-Orai1 WT before and after treatment with 1 μM thapsigargin (TG). The number of measured cells is indicated within each bar. (B,C) Confocal fluorescence images of representative cells expressing STIM1 (B) or STIM1 I409S (C) before and after treatment with 1 μM TG. CFP is shown in green, YFP in red. Scale bars are 5 µm.

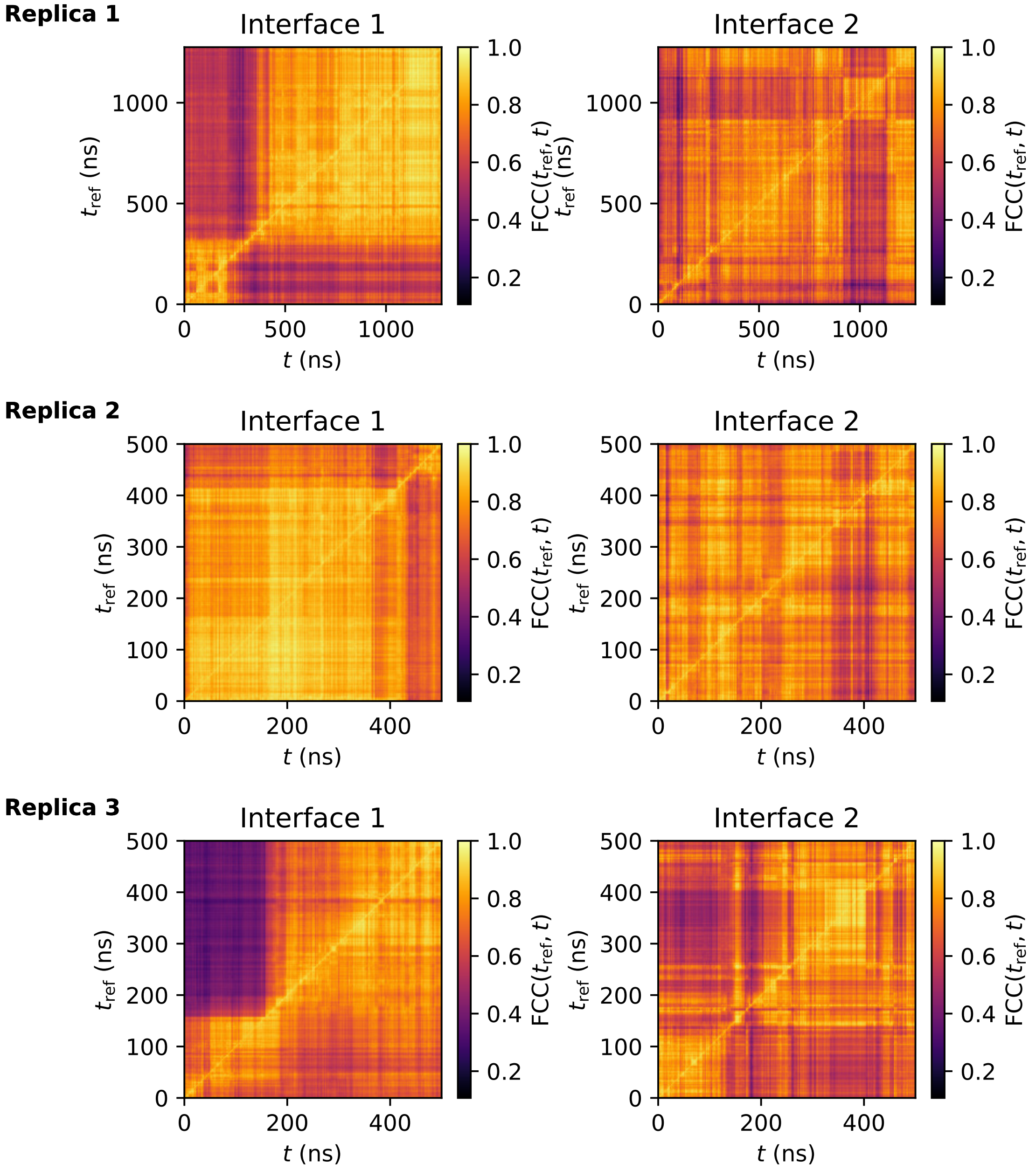

Figure S10: Fraction of common contact maps FCC(t_{ref}, t) for contacts between CC1α1 and CAD/SOAR in each replica of the dimeric model. Note that the CC1α1-CAD/SOAR binding interface in Replica 1, Interface 1 undergoes a clear transition within the first ≈ 500 ns as the domain-swapped dimeric structure equilibrates. By contrast, the transition found in Replica 3, Interface 1 at $t \approx 200$ ns corresponds to an overall *increase* in CC1α1-CAD/SOAR contacts with little to no *shift* of the binding configuration (the transition is visible only in the top half of the FCC map).

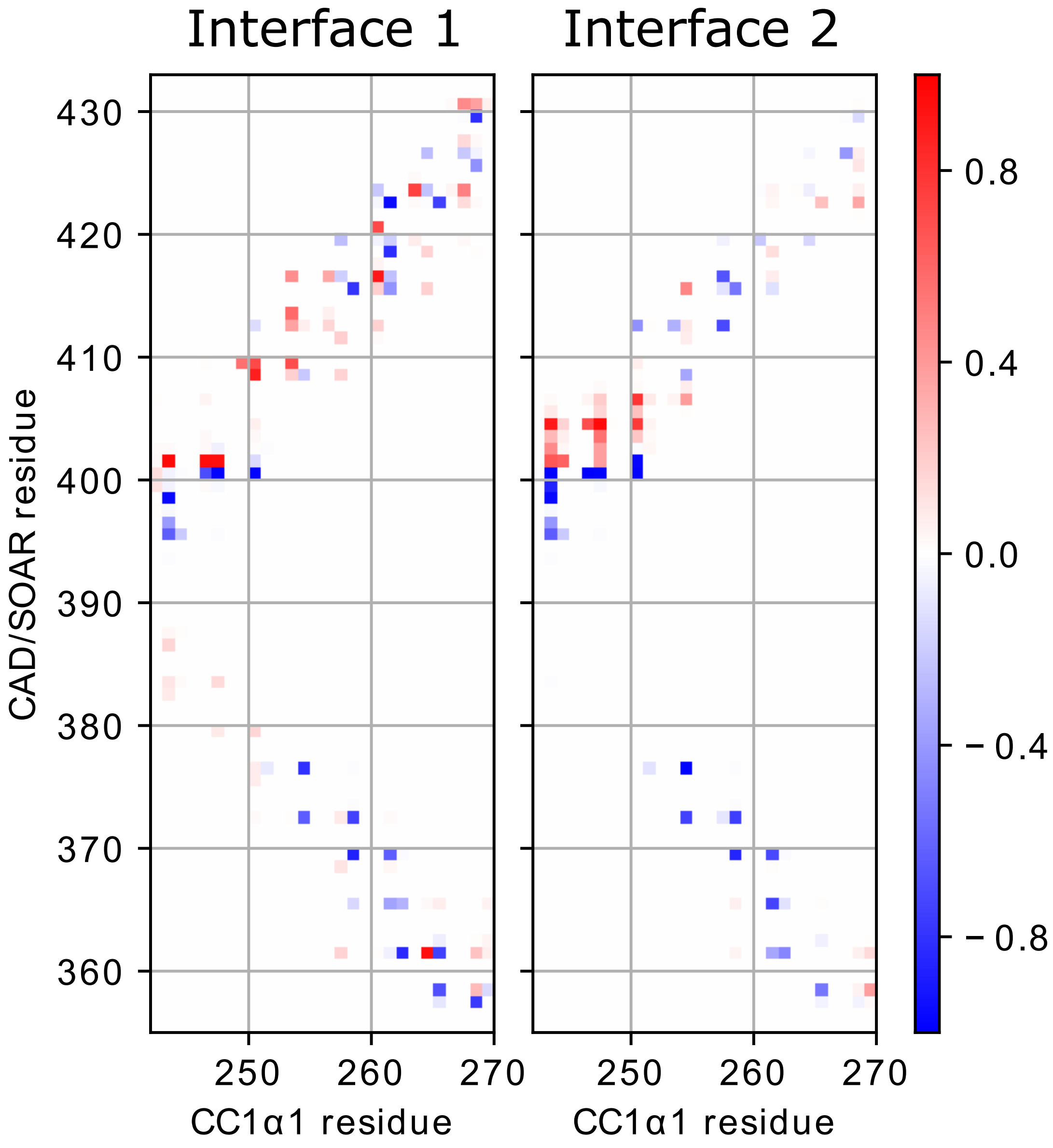

Figure S11: Comparison between CC1α1-CAD/SOAR binding configurations obtained for the monomeric and the dimeric models. For both interfaces present in the dimeric model, the change in contact frequency with respect to the monomeric model is shown. Red (blue) colors indicate larger (lower) contact frequencies compared to the monomeric model. Dimeric contact frequency was calculated using data from all three combined replicas.

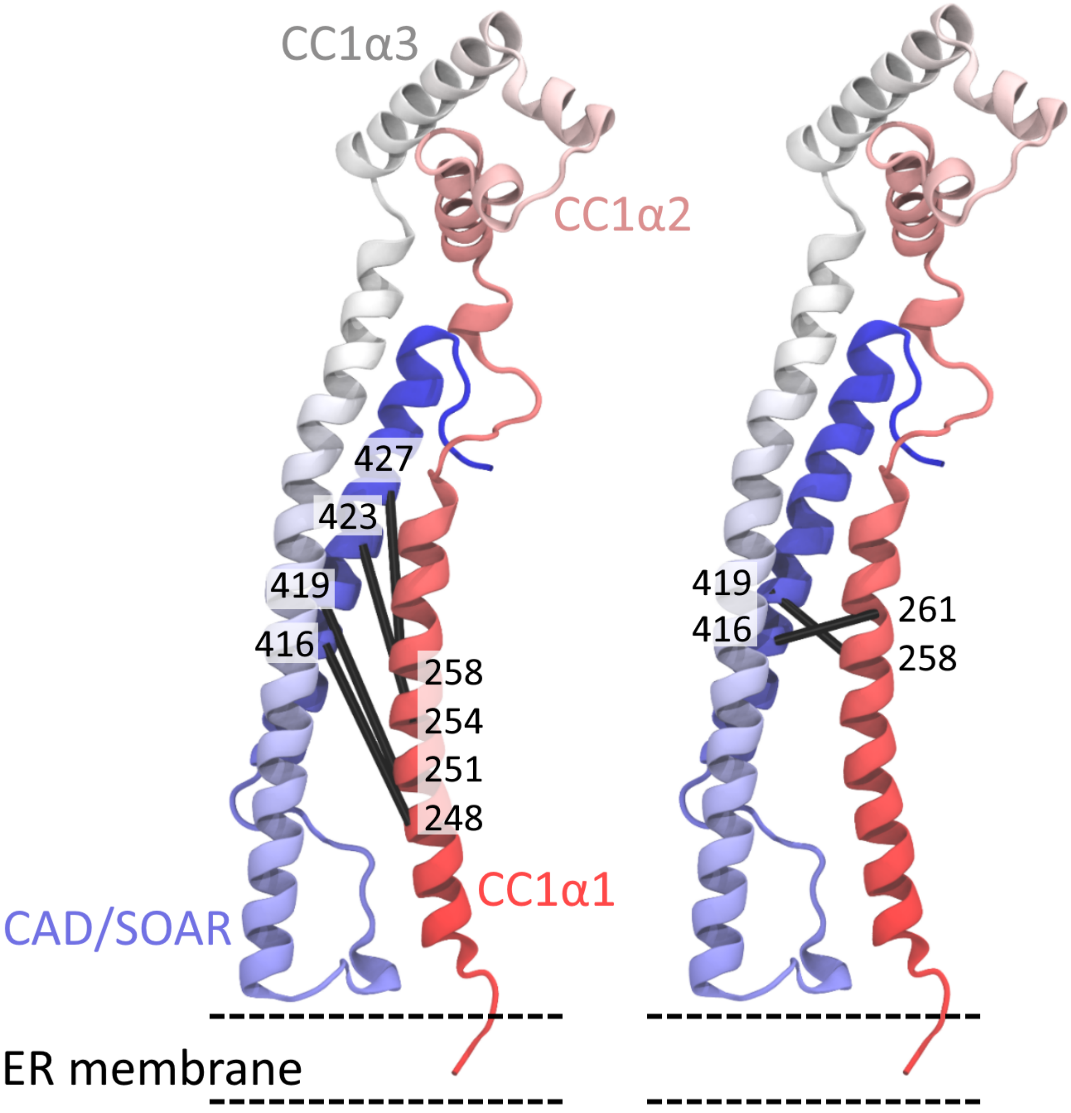

Figure S12: Model structure of the STIM1 quiescent state (residues 234-443). Residues connected by black lines in the left and right panels are proposed to be directly opposing binding partners according to references [1] and [2], respectively. Dashed lines mark the approximate position of the ER membrane.

#	Residue pair	smFRET distance (Å)	Mean simulated distance (Å)
1	239:239'	50	45.8
2	239:274	39	51.8
3	239:400	39	39.7
4	242:242'	48	37.2
5	242:363	38	41.9
6	242:378	35	34.8
7	242:389	42	33.1
8	242:400	36	36.7
9	242:400'	21	9.2
10	242:417	44	34.0
11	242:417'	25	29.7
12	242:431	48	47.5
13	242:431'	33	49.1
14	266:266'	41	30.5
15	266:389	52	53.9
16	274:274'	41	36.7
17	274:307	37	40.2
18	274:337	26	28.8
19	274:400	51	67.2
20	274:400'	46	50.9
21	274:417'	32	21.5
22	274:431	36	27.1
23	274:431'	25	11.6
24	298:298'	35	44.7
25	298:363	49	51.3
26	298:378	57	70.4
27	298:389	62	84.2
28	307:337	36	26.9
29	307:400	57	94.1
30	309:309'	25	61.2
31	312:312'	27	52.1
32	337:337'	26	29.7
33	337:363	34	41.3
34	337:378	43	61.9
35	337:389	55	77.1
36	337:431	30	27.3

Table S2: Residue pairs used for Figure 5B. Residues in the second STIM1 monomer are denoted by a prime. smFRET distances were taken from [1]. As in [1], 10 Å were subtracted from each smFRET-derived distance to account for fluorophore linkers. Simulated data refers to center of mass distances averaged over three independent MD replicas, each with a length of 500 ns.

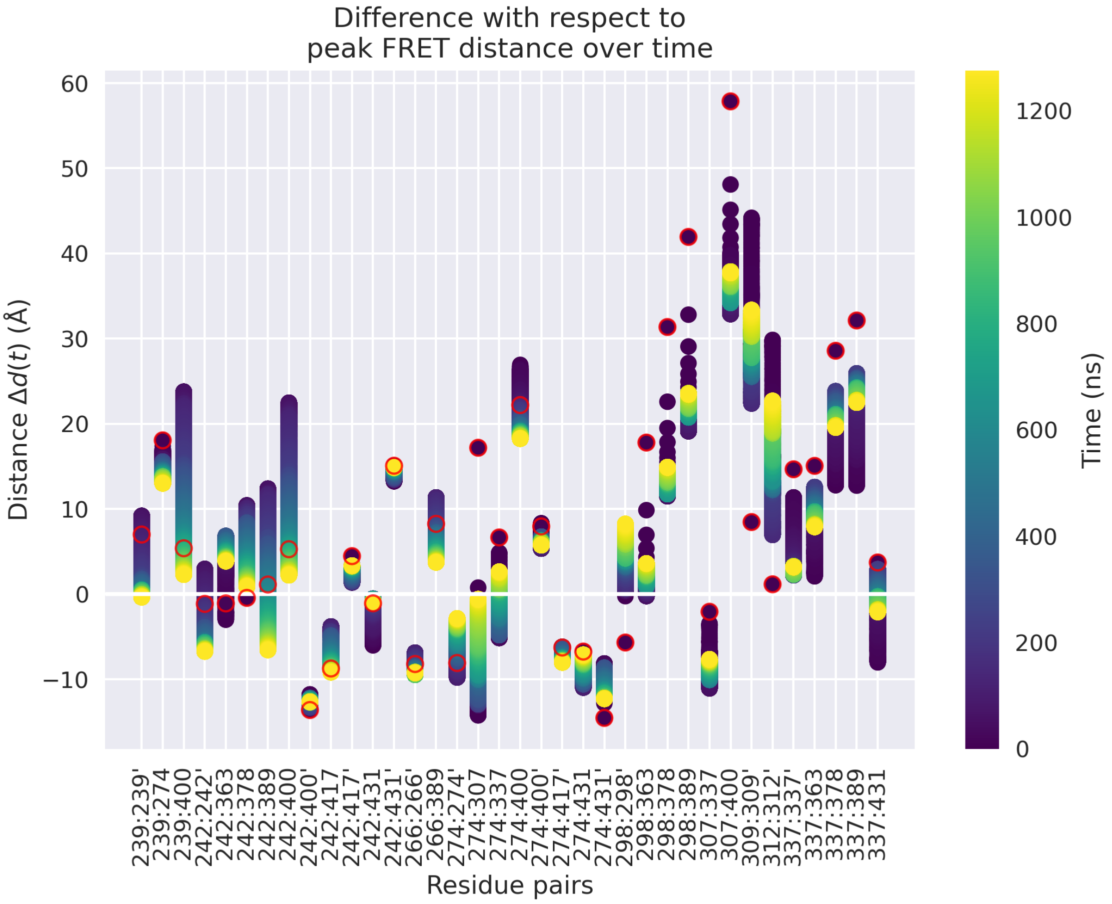

Figure S13: For each residue pair with available smFRET distance data, the difference Δd between our modelled distance d^{MD} and the experimentally determined distance d^{exp} is shown (time evolution is color-coded from blue to yellow). Initial distances $d^{\mathrm{MD}}(t=0)$ obtained from our docked model, before simulating molecular dynamics, are denoted by red circles. Modelled distance $\Delta d(t)$ is smoothed by a time average over all time steps $t_i < t$, i.e. $\Delta d(t) = \operatorname*{mean}_{t_i<t}\left(d^{\mathrm{MD}}(t_i) - d^{\mathrm{exp}}\right)$. For 27 out of 36 residue pairs, simulated distances relax towards d^{exp} over the course of the simulation. Note that those residue pairs where the deviation between simulated and experimental distances increases over the course of the simulation, 309:309' and 312:312', are situated at the tip of the CC1α1,2 hairpin, which is an especially mobile region (see Figure S15).

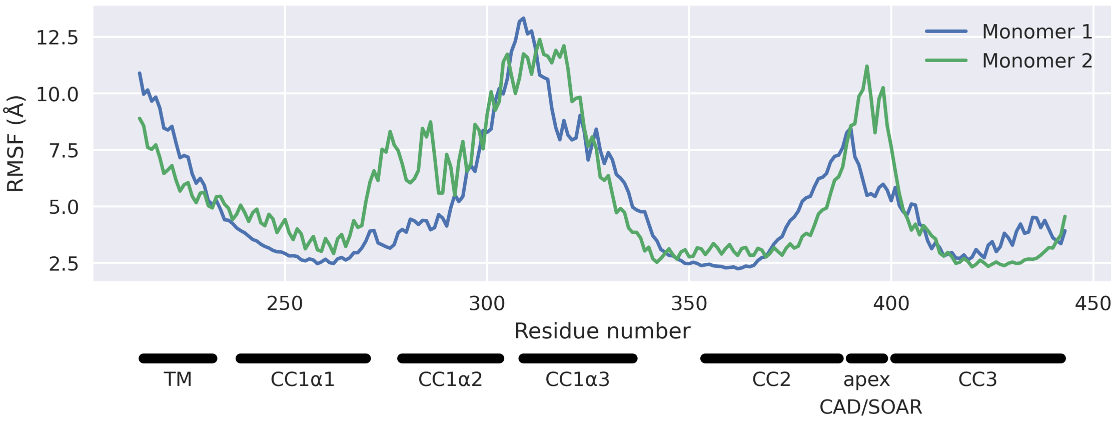

Figure S14: RMSF for Cα atoms in our dimeric STIM1 model. RMSF was calculated over concatenated data from 3 independent, superimposed 500 ns runs of unrestrained MD.

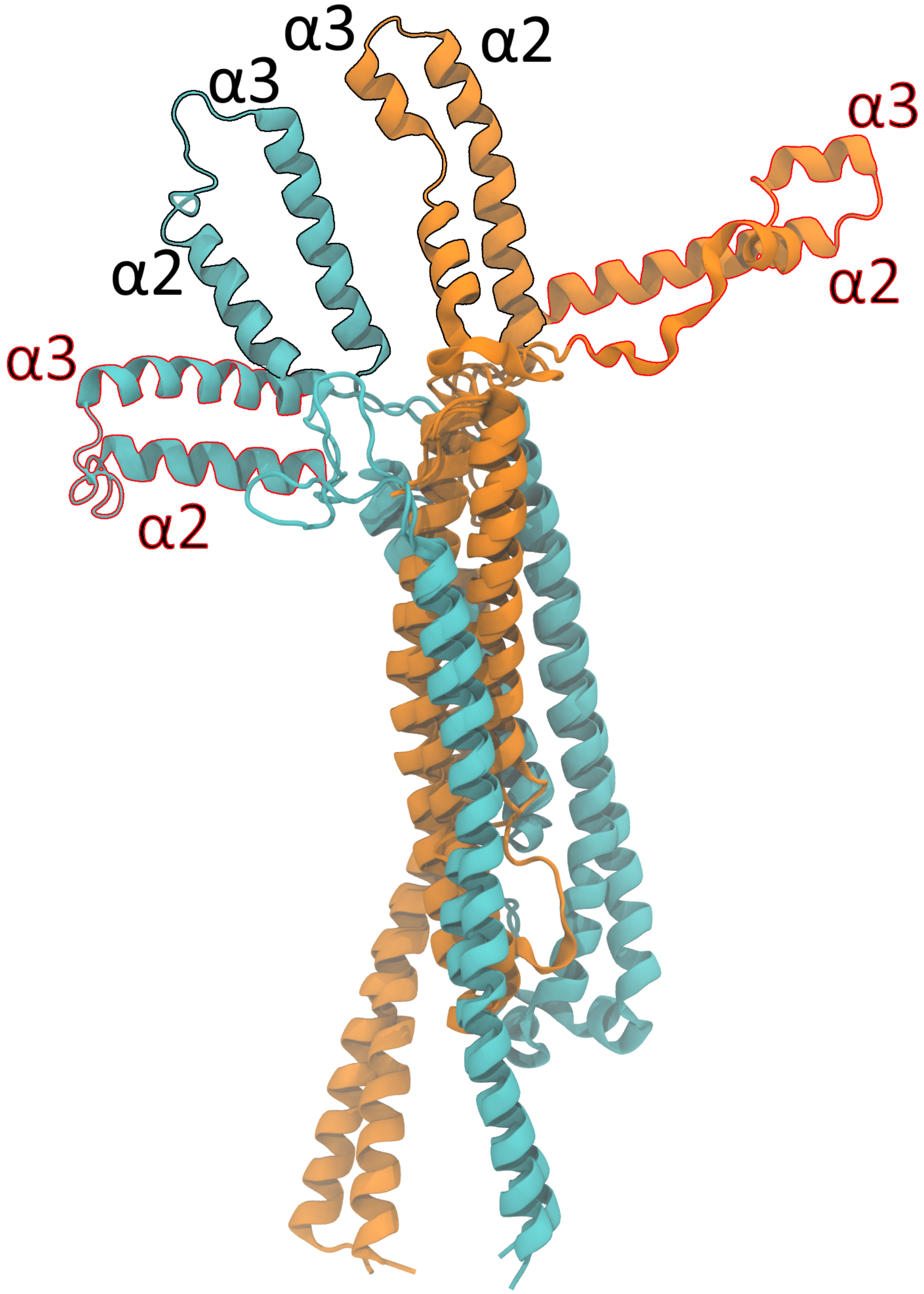

Figure S15: Illustration of the transition between a compact and splayed state of CC1α2/α3 observed over a timespan of about 200 ns in unrestrained MD. The compact and a splayed states are outlined in black and red, respectively. Orange and cyan structures correspond to the two monomers.

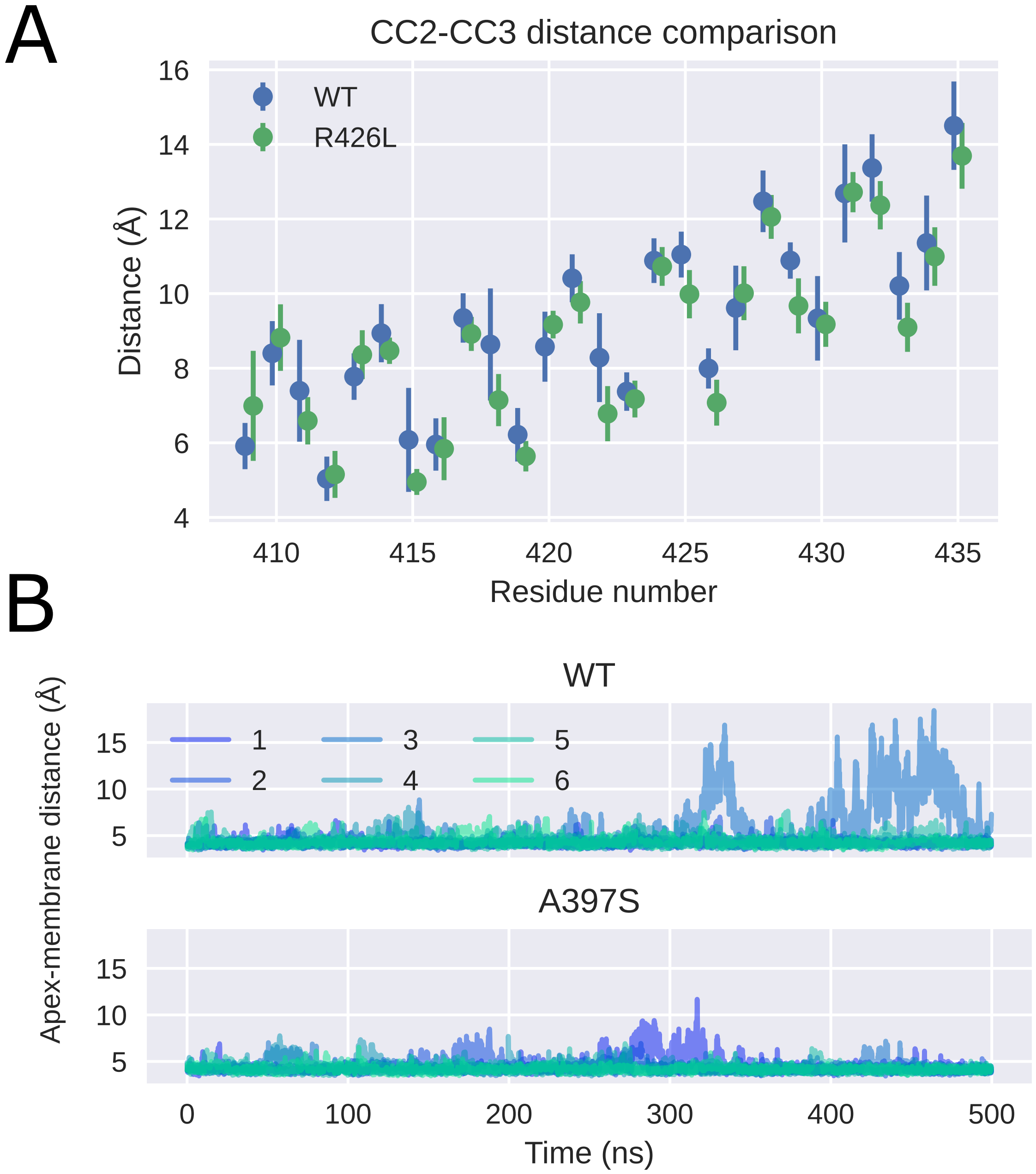

Figure S16: (A) CC2-CC3 distances for the WT and the R426L mutant simulated in the dimer model. For each residue in CC3, distances were calculated between Cα atoms, using the distance to the nearest residue in CC2. (B) CAD/SOAR-membrane distance for the dimeric WT and the A397S mutant, respectively. Distances were calculated as the closest interatomic distance between CAD/SOAR Cα and lipid phosphor atoms. They were recorded for both CAD/SOAR domains in three simulation replicas, resulting in six time series.

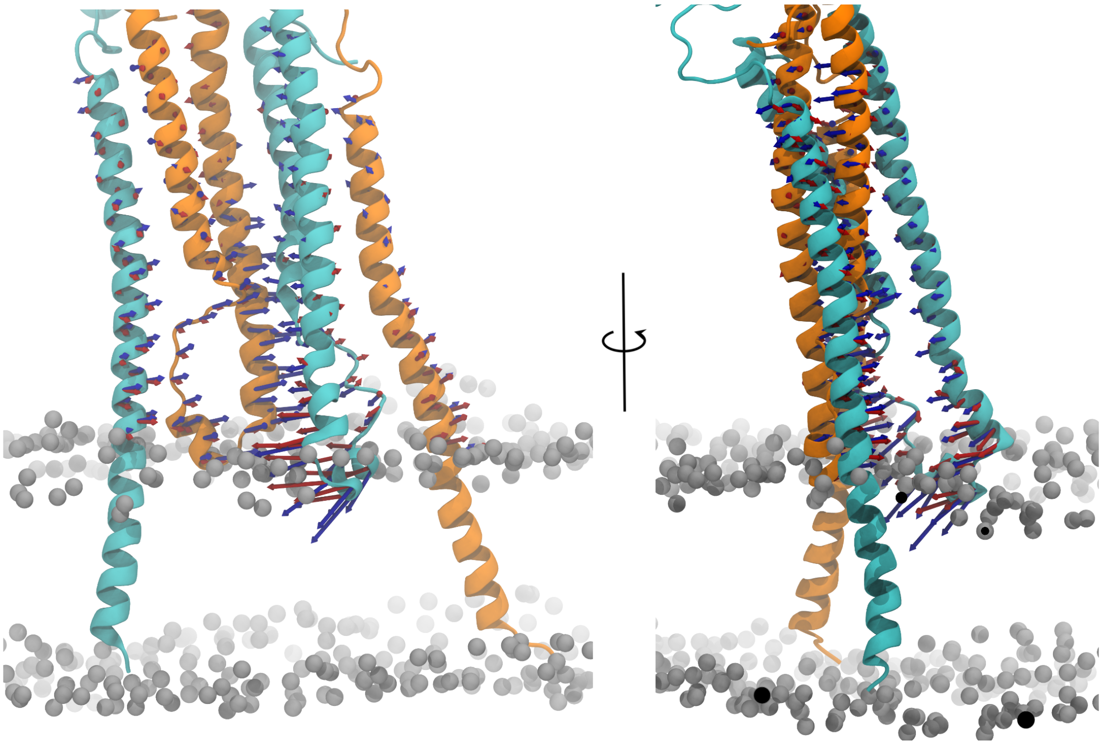

Figure S17: Overlay of the leading modes of motion obtained from a principal component analysis (PCA) of atomic fluctuations and an anisotropic network model (ANM) consisting of Cα atoms, respectively. Displacement vectors obtained from the PCA and ANM are shown in blue and red, respectively. Both methods indicate that the dominating deformation of the STIM1 tight, quiescent state dimer involves the two CAD/SOAR domains swinging out of the plane connecting the two CC1α1 helices in opposite directions.

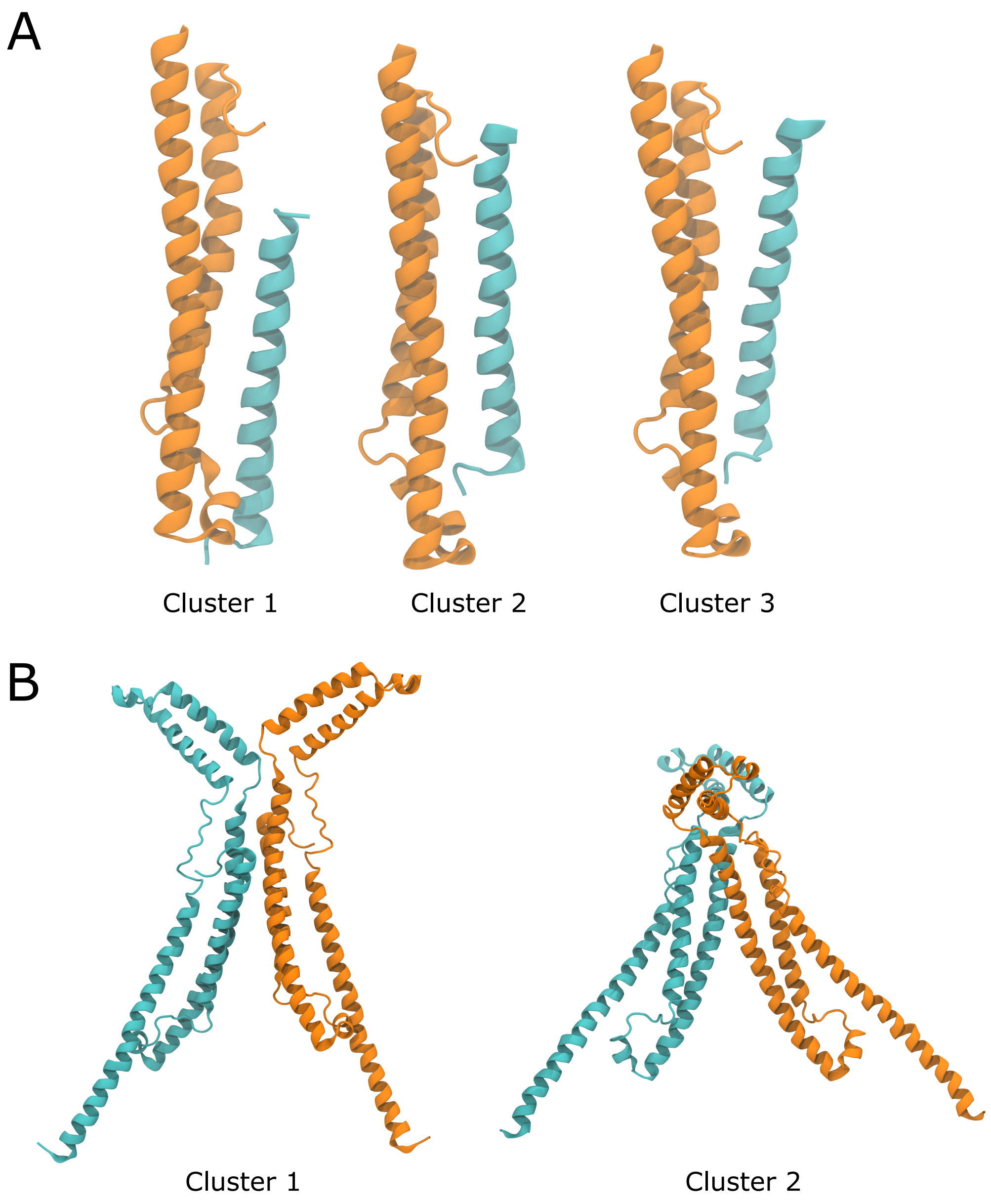

Figure S18: (A) Structures from the three best-scoring clusters obtained from docking CC1α1 and CAD/SOAR using HADDOCK. CAD/SOAR is shown in orange, CC1α1 in cyan. Docking scores for the three clusters were -85.7, -78.2 and -44.4. Our STIM1 monomeric model is based on Cluster 1. (B) Structures from the two best-scoring clusters obtained from docking two copies of our monomeric STIM1 model. Docking scores for the two clusters were -108.0 and -94.0. For the subsequent modeling of our domain-swapped dimer, we proceeded with Cluster 1.

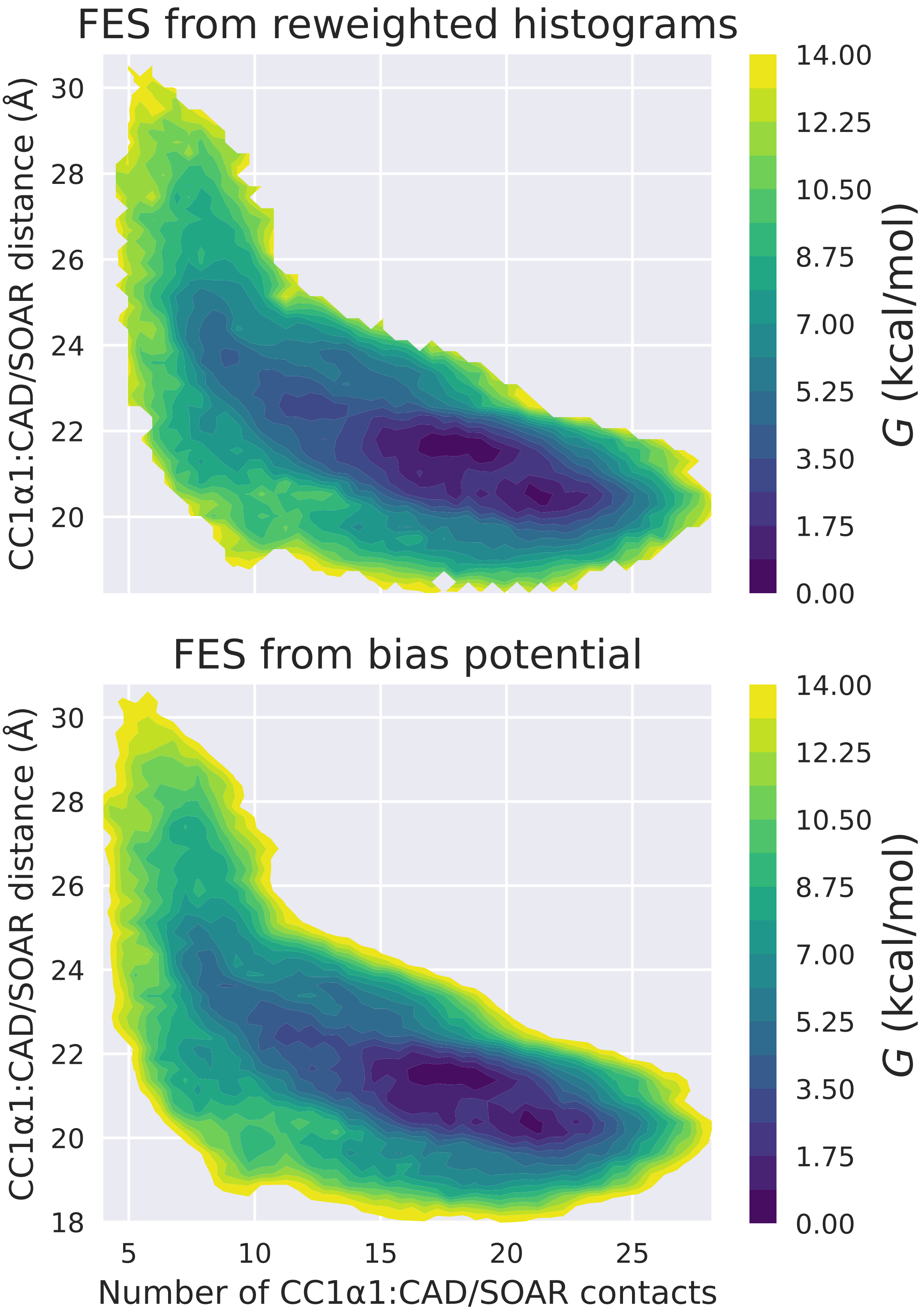

Figure S19: Exemplary free energy surface (FES) calculated with two different methods for the STIM1 WT. Top: FES calculated via the reweighted histogram of the biased collective variables. Bottom: FES calculated directly via the metadynamics bias potential.

Supplementary Appendix: STIM1:Orai1 cooperative binding model

In patch clamp experiments, one probes the free energy difference $\Delta G = G_{act} – G_{quies}$ between the activated and the quiescent STIM1 states, which can be dissected into three contributions,

$$\Delta G = \Delta G_{\text{unb}} + \Delta G_{\text{unb}\to\text{open}} + \Delta G_{\text{open}\to\text{act}}, \tag{S1}$$

where $\Delta G_{\text{unb}} + \Delta G_{\text{unb}\to\text{open}}$ is the free energy of STIM1 opening illustrated in Figure 6D and $\Delta G_{\text{open}\to\text{act}}$ describes the activation of Orai1. From our simulations we extract ΔG_{unb} rather than ΔG. We assume that mutations in the CC1α1-CAD/SOAR binding interface only affect the free energy of unbinding, ΔG_{unb}, i.e. $\Delta G_{\text{unb}\to\text{open}}$ and $\Delta G_{\text{open}\to\text{act}}$ are constant for different mutations (see Figure 6D).

The overall equilibrium constant of STIM1 activation may be decomposed according to

$$K_{\text{STIM1 open, act}} = K_{\text{STIM1 open}} \cdot K_{\text{STIM1 act}}, \tag{S2}$$

where the equilibrium constant of STIM1 opening/elongation reads

$$K_{\text{STIM1 open}} = \frac{[\text{STIM1}_{\text{open}}]}{[\text{STIM1}_{\text{quiesc}}]} \tag{S3}$$

$$= \exp\left(-\frac{\Delta G_{\text{unb}} + \Delta G_{\text{unb}\to\text{open}}}{RT}\right), \tag{S4}$$

and that of STIM1 activation by coupling to Orai1 is given as

$$K_{\text{STIM1 act}} = \exp\left(-\frac{\Delta G_{\text{open}\to\text{act}}}{RT}\right). \tag{S5}$$

The experimentally determined CRAC channel current before store depletion, $I(t = 0)$, is proportional to the concentration of bound STIM1-Orai1 complexes,

$$I(t = 0) \propto [\text{Orai1}\,\text{STIM1}^{(n)}_{\text{open}}] = K_{\text{STIM1 act}}[\text{Orai1}][\text{STIM1}_{\text{open}}]^n, \tag{S6}$$

where n denotes the cooperativity parameter [3–5], which takes into account that more than one STIM1 dimer binds to Orai1 and that successive binding events influence each other [6, 7]. By rewriting $[\text{STIM1}_{\text{open}}]$ as

$$[\text{STIM1}_{\text{open}}] = [\text{STIM1}_{\text{total}}] - [\text{STIM1}_{\text{quiesc}}] \tag{S7}$$

$$= [\text{STIM1}_{\text{total}}] - \frac{[\text{STIM1}_{\text{open}}]}{K_{\text{STIM1 open}}} \tag{S8}$$

$$= \frac{[\text{STIM1}_{\text{total}}]}{1 + 1/K_{\text{STIM1 open}}} \tag{S9}$$

we obtain

$$I(t = 0) = c_0 \cdot \left(\frac{1}{1 + c_1 e^{\Delta G_{\text{unb}}/RT}}\right)^n, \tag{S10}$$

where c_0 is a proportionality constant that also accounts for $K_{\text{STIM1 act}}$, $[\text{STIM1}_{\text{total}}]$ and $[\text{Orai1}]$ and $c_1 = \exp\left(\frac{\Delta G_{\text{unb}\to\text{open}}}{RT}\right)$.

Fitting our data on ΔG_{unb} and $I(t = 0)$ to equation (10) yields a cooperativity parameter of $n = 0.46 \pm 0.15$, indicating negative cooperativity (see Figure 6E). Furthermore, we obtain $\Delta G_{\mathrm{unb}\to\mathrm{act}} = -4.81 \pm 0.01$ kcal/mol, which suggests that at resting conditions ΔG_{unb} is comparable to $-\Delta G_{\mathrm{unb}\to\mathrm{act}}$ for mutants such as M244S ($\Delta G_{\mathrm{unb}}^{\mathrm{M244S}} = 7.67$ kcal/mol).

Fitting was performed with Orthogonal Distance Regression [8] using SciPy [9].

Supplementary Methods

Model construction

For docking CC1α1 (PDB id 6YEL [10]) to CAD/SOAR using the HADDOCK 2.4 webserver [11, 12], unambiguous restraints were added to reinforce pairing between BS^3 crosslinked residues: distances between residue pairs 413:243, 413:246, 386:238, 384:238 and 246:384 were restrained to values below 10 Å with default energy constant scaling for the four HADDOCK stages. Clustering was performed based on the fraction of native contacts. All other docking parameters were kept at their default values. To select the starting model for our MD simulations, clusters were sorted by the HADDOCK score of the best-scoring structure within each cluster (instead of their average score; see Figure S18A).

For the assignment of protonation states, we calculated electrostatic potentials using TAPBS [13]. Protonation states were assigned according to protonation patterns sampled using KARLSBERG 2.0 [14]. All titratable residues were found to occupy their standard protonation states, except for histidines, which showed varying patterns of protonated or neutral tautomeric states. The water solvating the final monomeric STIM1 model box was $160 \times 110 \times 95$ Å^3 in size, containing the protein, 51186 water molecules and 8 chloride counterions. To suppress rotation of our model, the positions of backbone atoms in CC1α1 were restrained during equilibration.

For the calculation of electrostatic interaction energies for CC1α1-CAD/SOAR salt bridges, we used gRINN [15] with the same settings employed in NAMD [16] non-bonded interaction calculation.

Metadynamics methods

The number of contacts was calculated with the `contacts` collective variable implemented in the colvars module [17], using parameters `cutoff` = 6 Å, `expNumer` = 6 and `expDenom` = 12. During out metadynamics runs, CC1α1 backbone positions were restrained to provide a firm basis from which CAD/SOAR is detached. All simulations were propagated up to a specific cutoff time, which was determined by calculating the number of contacts, N_c, between CC1α1 and CAD/SOAR and terminating the run once N_c fully zeroes out for at least 200 ps. This procedure ensures that all simulations stop just when the initial CC1α1-CAD/SOAR binding minimum is fully filled up by the bias potential. Secondly, since the contact frequency relies on the notion of a total run length t_c (see below), this cutoff scheme facilitates a consistent definition of t_c across all runs. Two-dimensional free energy surfaces were obtained by integrating out the contact collective variable. The CC1α1-CAD/SOAR binding free energy is then given by the "depth" of the free energy well, i.e. $G_{\min}$–G_{d_∞}, where d_∞ is a large, unsampled CC1α1-CAD/SOAR distance where the calculated free energy profile is flat.

Reweighting of non-biased observables was done using the balanced exponential reweighting scheme by Schäfer and Settanni [18], with weights

$$w^{\text{bex}} = \frac{e^{\beta V(s(r),t)}}{e^{\beta \langle V(s,t)\rangle}}, \tag{S11}$$

where $s(r)$ denotes a collective variable at point r in coordinate space, $V(s(r),t)$

is the time-dependent metadynamics bias potential, $\beta = \frac{1}{k_B T}$ and $\langle \cdot \rangle$ denotes the average over the collective variable s.

The biased contact frequency ω_{ij}^b was calculated directly from our metadynamics runs as

$$\omega_{ij}^b = \frac{\Delta t}{t_c} \sum_{t=0,\Delta t,2\Delta t,...}^{t=t_c} \theta(d_c - d_{ij}(t)), \tag{S12}$$

for a pair of binding residues i and j, one of which is in CC1α1 and one in CAD/SOAR. Using the weights w_t^{bex}, the reweighted contact frequency is given by

$$\omega_{ij} = \frac{\Delta t}{t_c} \sum_{t=0,\Delta t,2\Delta t,...}^{t=t_c} w_t^{\text{bex}} \theta(d_{\text{cut}} - d_{ij}(t)). \tag{S13}$$

Here, Δt is the time interval at which atomic coordinates were stored (20 ps), θ denotes the step function with cutoff distance d_c = 5 Å and $d_{ij}(t)$ is the lowest interatomic distance between residues i and j at frame t.

Dimeric model

After extending our monomeric STIM1 model by the trans-membrane domain, the extended model (residues 214-443) was dimerized using HADDOCK. Based on contact sites in the CAD/SOAR crystal (PDB id 3TEQ [19]), residues 346, 347, 350, 351, 353, 354, 357, 358, 361, 362, 365, 368, 425, 428, 429, 430, 432, 433, 436 and 437 were chosen as active residues. On grounds of Figure 5—figure supplement 2 and Figure 2B in reference [1], position 371 was added as a further active residue and the distance between residues 400 and 400' was restrained to values below 50 Å. To stabilize parallel orientation of the two monomers, the distance between residues 218 and 218' was restrained to values below 90 Å. All other docking parameters were set as described in section 4.1. Comparing with smFRET-derived distances reported in [1] showed that the angle between our two monomers needed to be corrected. To this end, we targeted and subsequently restrained distances between residue pairs 309:309', 312:312', 388:388', 389:389' and 399:399' using harmonic restraints with target distances of 35 Å, 37 Å, 48 Å, 46 Å, and 42 Å (derived from peak FRET values shown in Figure 2B in [1] and using Supplementary file 1 in [1]) while treating helical elements as rigid bodies by restraining dihedral angles [20]. From the 10 ns restrained MD run, we picked the frame with the lowest inter-monomeric interaction energy (calculated using CHARMM [21]). Using this structure, we assigned protonation states using TAPBS and KARLSBERG 2.0 as described above, but assigning a dielectric constant of 2 to a slab of 27 Å thickness to model the membrane as a separate dielectric medium. All titratable residues were found to occupy their standard protonation states, except for histidines, which showed varying patterns of protonated or neutral tautomeric states.

The anisotropic network model was constructed with ProDy [22] with a springs connecting all atoms within a 15 Å radius and a unit spring constant.

FRET Microscopy

The apparent FRET efficiency E_{app} was calculated after threshold determination and background signal subtraction. This was done on a pixel-to-pixel basis using a custom program [23] integrated into MATLAB (v7.11.0, The MathWorks, Inc., MA,

USA) [24] and implementing the method described in [25] with a microscope-specific constant G parameter of 2.75. The Pearson correlation coefficient (R value) was used to measure the strength of the linear association/co-localization between STIM1 and Orai1 variants, where a value of $R = 1$ signifies perfect positive correlation/co-localization.

Electrophysiology

For store-dependent currents, the initial current amplitude recorded during the voltage ramp applied immediately after achieving the whole-cell configuration was subtracted from all subsequent current amplitudes. For constitutively active currents, a current amplitude obtained during a voltage ramp applied after perfusion with 10 µM La^{3+} at the end of the experiment was used instead. Individual experiments were normalized by dividing all current amplitudes by the whole-cell capacitance, resulting in current density in picoampere per picofarad (pA/pF), to compensate for potential cell dimension-related effects and make individual experiments comparable. Individual time-series were aligned such that they reach maximum activation at the same time.

References

[1] S. van Dorp, R. Qiu, U. B. Choi, M. M. Wu, M. Yen, M. Kirmiz, A. T. Brunger, and R. S. Lewis, "Conformational dynamics of auto-inhibition in the er calcium sensor stim1," *eLife*, vol. 10, p. e66194, Nov 2021.

[2] G. Ma, M. Wei, L. He, C. Liu, B. Wu, S. L. Zhang, J. Jing, X. Liang, A. Senes, P. Tan, S. Li, A. Sun, Y. Bi, L. Zhong, H. Si, Y. Shen, M. Li, M. S. Lee, W. Zhou, J. Wang, Y. Wang, and Y. Zhou, "Inside-out Ca2+ signalling prompted by STIM1 conformational switch," *Nature Communications*, vol. 6, 2015.

[3] A. V. Hill, "The possible effects of the aggregation of the molecules of haemoglobin on its dissociation curves," *J. Physiol.*, vol. 40, pp. 4–7, 1910.

[4] N. Price, R. Dwek, R. Ratcliffe, and M. Wormald, *Principles and Problems in Physical Chemistry for Biochemists.* Oxford University Press, 2001.

[5] H. Bisswanger, *Multiple Equilibria*, ch. 1, pp. 7–58. John Wiley and Sons, Ltd, 2008.

[6] M. Yen and R. S. Lewis, "Cell Calcium Numbers count : How STIM and Orai stoichiometry affect store-operated calcium entry," *Cell Calcium*, vol. 79, no. November 2018, pp. 35–43, 2019.

[7] P. J. Hoover and R. S. Lewis, "Stoichiometric requirements for trapping and gating of stromal interaction molecule 1 (STIM1)," vol. 108, no. 32, 2011.

[8] P. T. Boggs and J. R. Donaldson, "Orthogonal distance regression," vol. 112, p. 186, 1989.

[9] P. Virtanen, R. Gommers, T. E. Oliphant, M. Haberland, T. Reddy, D. Cournapeau, E. Burovski, P. Peterson, W. Weckesser, J. Bright, S. J. van der Walt, M. Brett, J. Wilson, K. J. Millman, N. Mayorov, A. R. J. Nelson, E. Jones, R. Kern, E. Larson, C. J. Carey, İ. Polat, Y. Feng, E. W. Moore, J. VanderPlas, D. Laxalde, J. Perktold, R. Cimrman, I. Henriksen, E. A. Quintero, C. R. Harris, A. M. Archibald, A. H. Ribeiro, F. Pedregosa, P. van Mulbregt, and SciPy 1.0 Contributors, "SciPy 1.0: Fundamental Algorithms for Scientific Computing in Python," *Nature Methods*, vol. 17, pp. 261–272, 2020.

[10] P. Rathner, M. Fahrner, L. Cerofolini, H. Grabmayr, F. Horvath, H. Krobath, A. Gupta, E. Ravera, M. Fragai, M. Bechmann, T. Renger, C. Luchinat, C. Romanin, and N. Müller, "Interhelical interactions within the stim1 cc1 domain modulate crac channel activation," *Nature Chemical Biology*, vol. 17, pp. 196–204, Feb 2021.

[11] S. J. De Vries, M. Van Dijk, and A. M. Bonvin, "The HADDOCK web server for data-driven biomolecular docking," *Nature Protocols*, vol. 5, no. 5, pp. 883–897, 2010.

[12] G. van Zundert, J. Rodrigues, M. Trellet, C. Schmitz, P. Kastritis, E. Karaca, A. Melquiond, M. van Dijk, S. de Vries, and A. Bonvin, "The haddock2.2 web server: User-friendly integrative modeling of biomolecular complexes," *Journal of Molecular Biology*, vol. 428, no. 4, pp. 720–725, 2016. Computation Resources for Molecular Biology.

[13] G. Kieseritzky and E.-W. Knapp, "Optimizing pka computation in proteins with ph adapted conformations," *Proteins: Structure, Function, and Bioinformatics*, vol. 71, no. 3, pp. 1335–1348, 2008.

[14] B. Rabenstein and E.-W. Knapp, "Calculated ph-dependent population and protonation of carbon-monoxy-myoglobin conformers," *Biophysical Journal*, vol. 80, pp. 1141–1150, Mar 2001.

[15] O. Serçinoğlu and P. Ozbek, "GRINN: A tool for calculation of residue interaction energies and protein energy network analysis of molecular dynamics simulations," *Nucleic Acids Research*, vol. 46, no. W1, pp. W554–W562, 2018.

[16] J. C. Phillips, D. J. Hardy, J. D. C. Maia, J. E. Stone, J. V. Ribeiro, R. C. Bernardi, R. Buch, G. Fiorin, J. Hénin, W. Jiang, R. McGreevy, M. C. R. Melo, B. K. Radak, R. D. Skeel, A. Singharoy, Y. Wang, B. Roux, A. Aksimentiev, Z. Luthey-Schulten, L. V. Kalé, K. Schulten, C. Chipot, and E. Tajkhorshid, "Scalable molecular dynamics on cpu and gpu architectures with namd," *The Journal of Chemical Physics*, vol. 153, no. 4, p. 044130, 2020.

[17] G. Fiorin, M. L. Klein, and J. Hénin, "Using collective variables to drive molecular dynamics simulations," *Molecular Physics*, vol. 111, pp. 3345–3362, Dec 1 2013.

[18] T. M. Schäfer and G. Settanni, "Data Reweighting in Metadynamics Simulations," *Journal of Chemical Theory and Computation*, vol. 16, no. 4, pp. 2042–2052, 2020.

[19] X. Yang, H. Jin, X. Cai, S. Li, and Y. Shen, "Structural and mechanistic insights into the activation of Stromal interaction molecule 1 (STIM1)," *Proceedings of the National Academy of Sciences*, vol. 109, no. 15, pp. 5657–5662, 2012.

[20] W. Humphrey, A. Dalke, K. Schulten, L. Trabuco, and E. Villa, "VMD ssrestraints plugin 1.1," *Journal of Molecular Graphics*, vol. 14, pp. 33–38, 1996.

[21] B. R. Brooks, C. L. Brooks III, A. D. Mackerell Jr., L. Nilsson, R. J. Petrella, B. Roux, Y. Won, G. Archontis, C. Bartels, S. Boresch, A. Caflisch, L. Caves, Q. Cui, A. R. Dinner, M. Feig, S. Fischer, J. Gao, M. Hodoscek, W. Im, K. Kuczera, T. Lazaridis, J. Ma, V. Ovchinnikov, E. Paci, R. W. Pastor, C. B. Post, J. Z. Pu, M. Schaefer, B. Tidor, R. M. Venable, H. L. Woodcock, X. Wu, W. Yang, D. M. York, and M. Karplus, "Charmm: The biomolecular simulation program," *Journal of Computational Chemistry*, vol. 30, no. 10, pp. 1545–1614, 2009.

[22] S. Zhang, J. M. Krieger, Y. Zhang, C. Kaya, B. Kaynak, K. Mikulska-Ruminska, P. Doruker, H. Li, and I. Bahar, "ProDy 2.0: increased scale and scope after 10 years of protein dynamics modelling with Python," *Bioinformatics*, vol. 37, pp. 3657–3659, 04 2021.

[23] I. Derler, M. Hofbauer, H. Kahr, R. Fritsch, M. Muik, K. Kepplinger, M. E. Hack, S. Moritz, R. Schindl, K. Groschner, and C. Romanin, "Dynamic but not constitutive association of calmodulin with rat trpv6 channels enables fine tuning of ca2+-dependent inactivation," *The Journal of Physiology*, vol. 577, no. 1, pp. 31–44, 2006.

[24] MATLAB, *version 7.11.0*. Natick, Massachusetts: The MathWorks Inc., 2015.

[25] T. Zal and N. R. Gascoigne, "Photobleaching-corrected fret efficiency imaging of live cells," *Biophysical Journal*, vol. 86, no. 6, pp. 3923–3939, 2004.
